# Endosomal catabolism of phosphatidylinositol 4,5-bisphosphate is fundamental in building resilience against pathogens

**DOI:** 10.1093/procel/pwae041

**Published:** 2024-08-01

**Authors:** Chao Yang, Longfeng Yao, Dan Chen, Changling Chen, Wenbo Li, Hua Tong, Zihang Cheng, Yanling Yan, Long Lin, Jing Zhang, Anbing Shi

**Affiliations:** Department of Biochemistry and Molecular Biology, School of Basic Medicine, Tongji Medical College and State Key Laboratory for Diagnosis and Treatment of Severe Zoonotic Infectious Disease, Huazhong University of Science and Technology, Wuhan 430030, China; Department of Biochemistry and Molecular Biology, School of Basic Medicine, Tongji Medical College and State Key Laboratory for Diagnosis and Treatment of Severe Zoonotic Infectious Disease, Huazhong University of Science and Technology, Wuhan 430030, China; Department of Biochemistry and Molecular Biology, School of Basic Medicine, Tongji Medical College and State Key Laboratory for Diagnosis and Treatment of Severe Zoonotic Infectious Disease, Huazhong University of Science and Technology, Wuhan 430030, China; Department of Biochemistry and Molecular Biology, School of Basic Medicine, Tongji Medical College and State Key Laboratory for Diagnosis and Treatment of Severe Zoonotic Infectious Disease, Huazhong University of Science and Technology, Wuhan 430030, China; Department of Biochemistry and Molecular Biology, School of Basic Medicine, Tongji Medical College and State Key Laboratory for Diagnosis and Treatment of Severe Zoonotic Infectious Disease, Huazhong University of Science and Technology, Wuhan 430030, China; Department of Biochemistry and Molecular Biology, School of Basic Medicine, Tongji Medical College and State Key Laboratory for Diagnosis and Treatment of Severe Zoonotic Infectious Disease, Huazhong University of Science and Technology, Wuhan 430030, China; Department of Biochemistry and Molecular Biology, School of Basic Medicine, Tongji Medical College and State Key Laboratory for Diagnosis and Treatment of Severe Zoonotic Infectious Disease, Huazhong University of Science and Technology, Wuhan 430030, China; Department of Biochemistry and Molecular Biology, School of Basic Medicine, Tongji Medical College and State Key Laboratory for Diagnosis and Treatment of Severe Zoonotic Infectious Disease, Huazhong University of Science and Technology, Wuhan 430030, China; Department of Biochemistry and Molecular Biology, School of Basic Medicine, Tongji Medical College and State Key Laboratory for Diagnosis and Treatment of Severe Zoonotic Infectious Disease, Huazhong University of Science and Technology, Wuhan 430030, China; Department of Biochemistry and Molecular Biology, School of Basic Medicine, Tongji Medical College and State Key Laboratory for Diagnosis and Treatment of Severe Zoonotic Infectious Disease, Huazhong University of Science and Technology, Wuhan 430030, China; Department of Biochemistry and Molecular Biology, School of Basic Medicine, Tongji Medical College and State Key Laboratory for Diagnosis and Treatment of Severe Zoonotic Infectious Disease, Huazhong University of Science and Technology, Wuhan 430030, China; Cell Architecture Research Center, Huazhong University of Science and Technology, Wuhan 430030, China

**Keywords:** *C*. *elegans*, RAB-10/Rab10, UNC-16/JIP3, sorting endosome, EGL-8/PLC-β, NHR-25/NR5A1/2, PI(4,5)P2

## Abstract

Endosomes are characterized by the presence of various phosphoinositides that are essential for defining the membrane properties. However, the interplay between endosomal phosphoinositides metabolism and innate immunity is yet to be fully understood. Here, our findings highlight the evolutionary continuity of RAB-10/Rab10’s involvement in regulating innate immunity. Upon infection of *Caenorhabditis elegans* with *Pseudomonas aeruginosa*, an increase in RAB-10 activity was observed in the intestine. Conversely, when RAB-10 was absent, the intestinal diacylglycerols (DAGs) decreased, and the animal’s response to the pathogen was impaired. Further research revealed that UNC-16/JIP3 acts as an RAB-10 effector, facilitating the recruitment of phospholipase EGL-8 to endosomes. This leads to a decrease in endosomal phosphatidylinositol 4,5-bisphosphate (PI(4,5)P2) and an elevation of DAGs, as well as the activation of the PMK-1/p38 MAPK innate immune pathway. It is noteworthy that the dimerization of UNC-16 is a prerequisite for its interaction with RAB-10(GTP) and the recruitment of EGL-8. Moreover, we ascertained that the rise in RAB-10 activity, due to infection, was attributed to the augmented expression of LET-413/Erbin, and the nuclear receptor NHR-25/NR5A1/2 was determined to be indispensable for this increase. Hence, this study illuminates the significance of endosomal PI(4,5)P2 catabolism in boosting innate immunity and outlines an NHR-25-mediated mechanism for pathogen detection in intestinal epithelia.

## Introduction

Numerous investigations have been conducted to determine the role of p38 MAPK signaling in mammalian innate immunity, with a particular concern on its effect on the intestinal immune system (Cuadrado and Nebreda, 2010; Feng and Li, 2011). Similarly, studies on the *Caenorhabditis elegans* intestine have shown the importance of the PMK-1/p38 MAPK pathway in defending against pathogen infections (Aballay et al., 2003; Irazoqui et al., 2010; Kim and Ewbank, 2018; Kim et al., 2002; Sifri et al., 2003). Upon initiation of the innate immune response, phospholipase C β is activated to hydrolyze phosphatidylinositol 4,5-bisphosphate (PI(4,5)P2) into unsaturated diacylglycerols (DAGs) (Hodgkin et al., 1998; O’Donnell et al., 2018; Peterson et al., 2022; Ziegler et al., 2009). These DAGs, acting as secondary messengers, activate TPA-1/PKC-δ, leading to the activation of DKF-2/PKD. This culminates in the phosphorylation of TIR-1/SARM1, a Toll/interleukin-1 receptor domain protein that is a key component of the PMK-1/p38 MAPK pathway (Ren et al., 2009; Shirai and Saito, 2002; Ziegler et al., 2009).

Investigations into pathogen infection and defense mechanisms in mammals have unveiled the involvement of endosomal transport (Belabed et al., 2020; Stanley et al., 2014). Recycling endosomes are needed for sustaining the intracellular distribution of homeostatic through cargos sorting in mouse intestinal cells, which helps preserve mucosal tolerance to microbiota (Yu et al., 2014). In addition, late endosomes have been determined to be major regulators of the immune response, and the transportation of TLRs to late endosomes is a significant mechanism for restraining TLR self-recognition (Majer et al., 2017). As regulatory hubs of endosomal transport, Rabs have garnered significant attention in the context of pathogen infection and the preservation of intestinal tissue homeostasis. In the mammalian intestine, Rab7 has been shown to play a role in the assembly of Salmonella-containing vacuoles (D’Costa et al., 2015; Forbester et al., 2018; Mohapatra et al., 2019). Furthermore, the absence of Rab21 in *Drosophila* intestinal cells has been associated with an aberrant release of the proinflammatory cytokine Upd3 (Nassari et al., 2022). Of note, the delta-type opioid receptor, a member of the G protein-coupled receptor (GPCR) family, has been observed to interact with Rab10 (Degrandmaison et al., 2020). Also, Gαq, a regulator of innate immunity downstream of GPCR, was found to be present in endosomes, although the functional relationship between Gαq and Rab10 has yet to be determined (Daly and Plouffe, 2023).

Cellular membranes display an uneven distribution of seven phosphoinositides, produced by the phosphorylation of the hydroxyl groups at the 3-, 4-, and 5-positions of the inositol ring of phosphatidylinositol. PI(4,5)P2 is generated by phosphatidylinositol 4-phosphate 5-kinases and phosphatidylinositol 5-phosphate 4-kinase, which phosphorylate PI(4)P and PI(5)P, respectively (Di Paolo and De Camilli, 2006). Besides the plasma membrane, PI(4,5)P2 metabolism also occurs in sorting endosomes and recycling endosomes further along the pathway (Shi et al., 2012; Chen et al., 2018; Farmer et al., 2021). By influencing the recruitment of regulatory proteins with varying affinities for phosphoinositides, the metabolic activity of PI(4,5)P2 can thus modulate endosomes functionality (Chen et al., 2018; Farmer et al., 2020; Gao et al., 2020; Liu et al., 2018; Shi et al., 2012; Zhang et al., 2023). It is worth noting that the metabolic breakdown of PI(4,5)P2 is highly significant for specific physiological processes. Evidence has shown that eliminating PI(4,5)P2 from endocytosed vesicles is essential to ensure subsequent endosomal transports (Donaldson, 2005; Grant and Donaldson, 2009; Naslavsky et al., 2004).

RAB-10/Rab10 is a critical regulator in the endocytic recycling within epithelia (Babbey et al., 2006; Chua and Tang, 2018; Etoh and Fukuda, 2019; Lazo and Schiavo, 2023; Nakamura et al., 2020; Schuck et al., 2007). In the intestine of *C*. *elegans*, RAB-10 exhibits localization in sorting endosomes, with its activity being orchestrated by LET-413/Erbin and DENN-4/GEF (Liu et al., 2018). The facilitation of endosomal membrane budding is a notable function of RAB-10, achieved by its ability to bridge endosomal PI(4,5)P2 and actin filaments through its effector EHBP-1/Ehbp1 (Wang et al., 2016). Additionally, RAB-10 is implicated in the regulation of endosomal PI(4,5)P2 level (Shi et al., 2012). The abnormally increased endosomal PI(4,5)P2 in RAB-10-deficient animals can be, at least in part, attributed to the impeded endosomal recruitment of CNT-1/ARF-6-GAP. This leads to the sustained activation of ARF-6, perpetuating the endosomal recruitment of PPK-1/PI4P-5 kinase for PI(4,5)P2 synthesis (Shi et al., 2012). It is noteworthy that the elevation of endosomal PI(4,5)P2 due to RAB-10 depletion is much more pronounced than that observed in CNT-1-depleted animals, suggesting that RAB-10 is also involved in PI(4,5)P2 metabolism through additional pathways, which requires further exploration.

JIPs are characterized by their capacity to interact with multiple kinases in the c-Jun N-terminal kinase (JNK) pathway (Whitmarsh, 2006). Of the four mammalian JIPs (JIP1, JIP2, JIP3, and JIP4), JIP3 and JIP4 exhibit a shared structural domain composition (Whitmarsh, 2006). Initially discovered in *Drosophila* as Sunday Driver (SYD), UNC-16/JIP3 has been demonstrated to function as a scaffolding protein capable of binding to both kinesin-1 and dynein (Arimoto et al., 2011; Byrd et al., 2001; Sun et al., 2011). Moreover, JIP3 has been implicated in regulating axonal transport through Rab5-positive endosomes (Deinhardt et al., 2006). Consistent findings in *C*. *elegans* showed that the absence of UNC-16 results in the buildup of RAB-5-containing compartments at presynaptic terminals, indicating that UNC-16 could be a negative regulator of RAB-5 (Brown et al., 2009). Investigations into ADP-ribosylation factor 6 (Arf6) provided further insight into the engagement of JIP3/JIP4 in endosomal trafficking. These inquiries have shown that JIP3/JIP4 functions as an effector of Arf6, regulating the movement of recycling endosomes during cytokinesis (Montagnac et al., 2009). Studies conducted more recently have broadened the functional range of JIP3/JIP4 to include lysosomal processes. In primary mouse astrocytes, LRRK2 recruits JIP4 to lysosomes through phosphorylation of Rab10, thereby enabling the release of lumenal contents from lysosomes (Bonet-Ponce et al., 2020). Subsequent proteomic research has corroborated this regulatory relevance, exhibiting a physical interaction between JIP3/JIP4 and Rab10 (Dumrongprechachan et al., 2022).

Here, our study revealed that RAB-10 is a crucial regulator of innate immunity within the intestinal epithelium of *C*. *elegans*, as its activity was increased during *P*. *aeruginosa* infection. Then, we identified UNC-16 as a RAB-10 effector situated on sorting endosomes, and the depletion of UNC-16 was accompanied by a decrease in DAGs and a weakened infection response. Through further screening and biochemical analysis, we established that UNC-16 forms dimers and functions as a potential scaffold protein for the endosomal recruitment of EGL-8, which acts as the primary phospholipase for PI(4,5)P2 hydrolysis and DAGs formation on endosomes, thereby activating the PMK-1/p38 MAPK innate immune pathway. Additionally, we determined that the augmented RAB-10 activity during *P*. *aeruginosa* infection was attributed to an upsurge in LET-413 expression, enabled by the nuclear receptor family transcription factor NHR-25. Together, our study illuminated an unprecedented regulatory mechanism for innate immunity in the intestine, underscoring the fundamental role of endosomal PI(4,5)P2 catabolism in innate immune efficacy, and expanding our knowledge of the p38 MAPK pathway.

## Results

### RAB-10 is indispensable for pathogen-induced innate immune responses in the intestine

**Figure 1.**
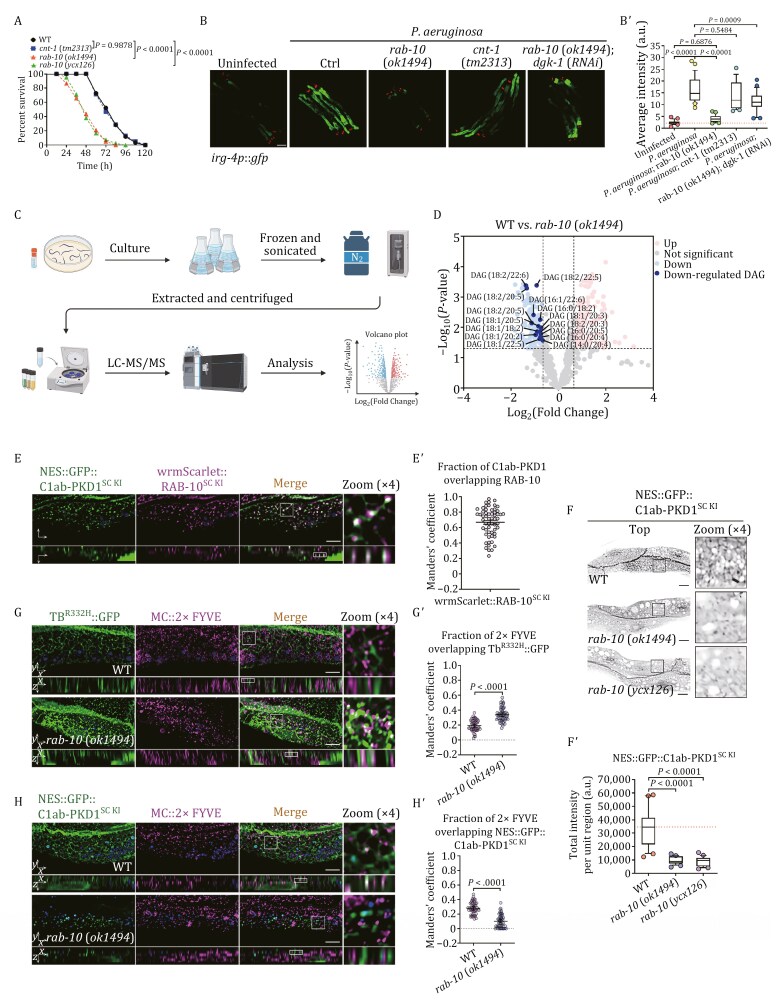
RAB-10 is necessary for pathogen-induced innate immune responses in the intestine, and its deficiency leads to a decline of DAGs in endosomes. (A) Survival of wild-type, *rab-10*(*ok1494*), *cnt-1*(*tm2313*), and *rab-10*(*ycx126*) animals exposed to *P*. *aeruginosa*. The *P*-values for the comparisons are indicated (log-rank test). Sample sizes and mean lifespan are shown in Table S2. (B and Bʹ) Confocal images of wild-type, *rab-10*(*ok1494*), and *cnt-1*(*tm2313*) animals expressing the transcriptional reporter *irg-4p*::*gfp* exposed to the indicated conditions. Box and whiskers plots (*n* = 25 animals): 10th–90th percentile; dots, outliers; midline, median of *wild-type* animals; boundaries, quartiles. The *P*-values for the comparisons are indicated (one-way ANOVA with Tukey’s multiple comparisons test). Scale bar, 100 µm. (C) A diagram showcases the application of LC–MS/MS in lipidomics studies of *C*. *elegans*. (D) A volcano plot illustrating the differential metabolite analysis between *rab-10*(*ok1494*) and wild-type animals is presented. Each point in the volcano plot represents a metabolite. The horizontal coordinates represent the fold change of the group comparing each substance (log_2_(fold change)), and the vertical coordinates represent the *P*-value of the *t*-test (−log_10_(*P*-value)). The raw data is filtered and categorized at *P* < 0.05, with a log_2_fold change >0.65 or <−0.65. Metabolites in the dataset that are significantly up-regulated are shown in pink, those significantly down-regulated are shown in blue, those that are not significantly different are shown in gray, and the DAGs annotated as significantly down-regulated are in dark blue. See also Table S1. (E and E´) Confocal images showing the colocalization (upper panels: Z-axis focal plane; lower panels: Y-axis focal plane) between NES::GFP::C1ab-PKD1^SC KI^ (SC KI: CRISPR/Cas9 single-copy transgene knock-in) and wrmScarlet::RAB-10^SC KI^ in the intestinal cells. The DAPI channel (blue color) indicates broad-spectrum intestinal autofluorescence. Manders’ coefficients were calculated using the z-stack confocal slices. The data is the mean with 95% confidence intervals (*n* = 9 animals). Scale bar, 10 μm. (F and Fʹ) Confocal images (top, focal plane near the basolateral membrane) showing the localization of NES::GFP::C1ab-PKD1^SC KI^ in wild-type, *rab-10*(*ok1494*), and *rab-10*(*ycx126*) animals. Box and whiskers plots (*n* = 24 regions): 10th–90th percentile; dots, outliers; midline, median of wild-type; boundaries, quartiles. The *P*-values for the comparisons are indicated (one-way ANOVA with Tukey’s multiple comparisons test). Scale bars, 10 μm. (G–Hʹ) Confocal images showing the colocalization (upper panels: Z-axis focal plane; lower panels: Y-axis focal plane) between mCherry::2xFYVE and Tubby-PH^(R332H)^::GFP or NES::GFP::C1ab-PKD1^SC KI^ in the intestinal cells. The DAPI channel (blue color) indicates broad-spectrum intestinal autofluorescence. Manders’ coefficients were calculated using the z-stack confocal slices. The data are the mean with 95% confidence intervals (*n* = 9 animals). The *P*-values for the comparisons are indicated (two-tailed, unpaired *t*-test). The scale bars are 10 μm.

In mouse macrophages, Rab10 promotes TLR4 signaling by enabling the delivery of TLR4 to the plasma membrane (Wang et al., 2010). Following TLR4 stimulation, the relocation of tumor necrosis factor from recycling endosomes to the plasma membrane is also observed (Stanley et al., 2014). To comprehend the conservative involvement of RAB-10/Rab10 in countering pathogens, a “slow-kill” assay was conducted in *C*. *elegans*, and the survival of the animals (*n* = 40) was observed at 12-hour intervals (Kirienko et al., 2014; Tan et al., 1999). Our results revealed a considerable decrease in the survival rate of *rab-10*(*ok1494*) mutant animals when exposed to *P*. *aeruginosa* strain PA14 (Fig. 1A), suggesting that the lack of RAB-10 increased susceptibility to infection. To assess the immune efficacy of nematodes, a transgenic strain expressing an intestinal *irg-4p*::*gfp* was deployed (Kawli et al., 2010; Peterson et al., 2023). Notably, the expression of *irg-4p*::*gfp* was upregulated upon *P. aeruginosa* infection (Fig. 1B and 1Bʹ), yet this response was not present when RAB-10 was absent, signifying the evolutionary preservation of RAB-10/Rab10’s participation in innate immunity. In addition, such results have generated curiosity as to how RAB-10 manages the innate immune response in metazoans without the Toll pathway.

Evidence from animal studies has demonstrated that the intestinal epithelium is essential for protecting against environmental pathogen infections (Cesar Machado and da Silva, 2016; Egge et al., 2019; Ma et al., 2020; Walker et al., 2014). In *C*. *elegans*, the p38 MAPK pathway is triggered in the intestine, granting it innate immunity against pathogens (Peterson et al., 2022; Zarate-Potes et al., 2020). To ascertain the importance of intestinal RAB-10 in the *P*. *aeruginosa* infection response, we utilized a CRISPR/Cas9-based tissue-specific approach to generate a strain, *rab-10*(*ycx126*), in which RAB-10 was selectively inactivated in the intestine. Our results indicated that *rab-10*(*ycx126*) animals had a comparable decline in survival rate to that of *rab-10*(*ok1494*) mutants (Fig. 1A), affirming that the intestine is the predominant contributor to the infection response. Of note, a plethora of research has demonstrated the significant role of RAB-10 during the recycling transport (Chen et al., 2006; Shi et al., 2012; Zhang et al., 2023), which brings to light that the impairment of basolateral recycling could be a contributing factor to the infection response defects. AMPH-1 is thought to recognize the curvature of endosomal membranes, thus promoting tubule formation and coordinating basolateral recycling (Liu and Grant, 2015; Pant et al., 2009). However, the survival rate of animals infected with *P*. *aeruginosa* was not evidently impacted by the absence of AMPH-1 (Fig. S1A), suggesting that the decrease in infection response caused by RAB-10 deficiency is not due to impaired recycling transport. This is corroborated by the fact that the lack of another basolateral recycling regulator, ARF-6/Arf6, did not worsen the survival of animals infected with pathogens (Fig. S1A). It should also be noted that ARF-6 promotes PI(4,5)P2 production on the plasma membrane and recycling endosome, with PPK-1/PI4P-5 kinase acting as a catalyst (Chen et al., 2018; Donaldson, 2003; Shi et al., 2012). Hence, our findings also suggest that the diminished pathogen resistance is unlikely to be caused by impaired anabolism of PI(4,5)P2.

### RAB-10 deficiency leads to a decline of DAGs in endosomes

Our evidence thus far has demonstrated that RAB-10 does not appear to influence pathogen resistance by managing basolateral recycling. Additionally, research has shown that RAB-10 is not responsible for the apical delivery of proteins related to pathogen resistance in the intestine (Wang et al., 2022). Therefore, it can be inferred that the anti-pathogen effects of RAB-10 could be achieved through other non-transport processes. Previous studies have revealed that the level of endosomal PI(4,5)P2 in RAB-10-deficient animals is abnormally high, and this is partially due to CNT-1/ARF-6-GAP, a RAB-10 effector, not being properly situated in the sorting endosome, thus resulting in an excessive activation of ARF-6 and an over-engagement of PPK-1 (Shi et al., 2012). Nevertheless, we found no substantial change in animal survival rate or infection response in CNT-1-deficient animals (Fig. 1A–Bʹ), corroborating that variations in the synthesis of endosomal PI(4,5)P2 do not have a considerable effect on innate immunity. It is worth noting that the increased levels of PI(4,5)P2 in RAB-10-deficient animals are remarkable, while the absence of CNT-1 is not able to account for this occurrence fully (Shi et al., 2012). Therefore, it is conceivable to postulate that the decreased hydrolysis of endosomal PI(4,5)P2 could also be associated with the deficiency of RAB-10, thus likely leading to a reduction of DAGs production. To test this speculation, we looked into whether DGK-1 knockdown could improve the expression of *irg-4p*::*gfp* in *rab-10* mutant animals. DGK-1/DGKs (diacylglycerol kinases) has been reported to phosphorylate DAGs and converts them into phosphatidic acid (Chen et al., 2023). Indeed, the lack of upregulation of *irg-4p*::*gfp* upon infection was rectified by the loss of DGK-1 (Fig. 1B and 1Bʹ). Additionally, DAG can be metabolized through three alternative pathways (Carrasco and Merida, 2007). First, DAG is capable of being converted into phosphatidylethanolamine (PtdEtn) and phosphatidylcholine (PtdCho) through the catalytic action of choline/ethanolamine phosphotransferase (CEPT1) and choline phosphotransferase (CPT1). However, there are currently no documented homologs of these two enzymes in the worm. Additionally, DAG can undergo esterification to form triacylglycerol, a reaction facilitated by diacylglycerol acyltransferase (DGAT). There are two main types of DGAT, namely DGAT1 and DGAT2. In *C*. *elegans*, DGAT1 has only one homolog, MBOA-2, while DGAT2 has four homologs: DGAT-2, K07B1.4, DGTR-1, and Y53G8B.2 (Yang et al., 2020). Furthermore, diacylglycerol can serve as a substrate for diacylglycerol lipases (DAGLs), which catalyze the hydrolysis of the fatty acid at position 1 or 2, resulting in the formation of monoacylglycerol. DAGL-1 is the identified homolog of DAGL in *C*. *elegans* (Lin et al., 2014). We examined the tissue expression profiles of these genes in WormBase (Version: WS292), and found that only DGAT-2 and K07B1.4 exhibit distinct expression in intestinal cells. We further investigated whether the depletion of DGAT-2 and K07B1.4 could also enhance the expression of *irg-4p*::*gfp* in *rab-10* mutants. By knocking down the expression of DGAT-2 and K07B1.4 through RNAi, we observed a reversal in the deficiency of *irg-4p*::*gfp* upregulation during infection (Fig. S1B and S1Bʹ). Together, these findings suggest that the attenuated innate immunity in RAB-10-deficient animals is likely due to a decrease in DAGs.

Furthermore, we aimed to investigate the intestinal DAG variations through lipidomics analysis. However, the *rab-10*(*ycx126*) animals show an efficiency of approximately 30% knockout per generation, which presents a challenge in effectively detecting changes in DAGs and results in notable variability in replicates. The intestine, a major organ in *C*. *elegans*, accounts for about one-third of the animal’s total somatic mass (McGhee, 2007). Differences in biomolecular composition within intestinal tissue can often be detected through whole-animal analyses, even in the absence of similar alterations in other tissues. Therefore, we selected to conduct targeted lipidomics on *rab-10*(*ok1494*) animals (*n* = 5) to assess changes in DAGs levels (Fig. 1C). Our findings indicated 283 types of lipids with abundance changes (*P < *0.05 and log_2_(fold change)* > *0.65 or < − 0.65), 127 of which were upregulated and 156 downregulated (Table S1). Notably, 15 types of DAG isomers were downregulated (Fig. 1D), mostly the polyunsaturated forms that serve as intracellular signals (Hodgkin et al., 1998).

By fusing the C1ab domain of PKD1, which has a high affinity for DAGs, with GFP containing the nuclear export signal (NES) (Kim et al., 2011; Nix and Beckerle, 1997), we generated a strain expressing NES::GFP::C1ab-PKD1^SC KI^ (SC KI: CRISPR/Cas9 single-copy transgene knock-in) to assay the distribution of DAGs in a living animal. Notably, there was a significant overlap between C1ab-PKD1^SC KI^ and the endosomal wrmScarlet::RAB-10^SC KI^ in intestinal cells (Fig. 1E and 1Eʹ). Moreover, in accordance with the lipidomics results, C1ab-PKD1^SC KI^ labeling on the punctate structures was reduced in both *rab-10*(*ok1494*) and *rab-10*(*ycx126*) mutant animals (Fig. 1F and 1Fʹ). In contrast, PI(4,5)P2 biosensor Tubby-PH^(R332H)^ was observed to accumulate in RAB-10-deficient cells, occurring more often in 2xFYVE-labeled early/sorting endosomes (Fig. 1G and 1Gʹ). Together with the reduced colocalization of C1ab-PKD1^SC KI^ with 2xFYVE in *rab-10*(*ok1494*) mutants (Fig. 1H and 1Hʹ), these results indicate that the lack of RAB-10 leads to a concurrent accumulation of PI(4,5)P2 and decrease of DAGs on sorting endosomes. Moreover, these findings suggest that the decline in *P*. *aeruginosa* infection response due to the absence of RAB-10 is likely associated with the impaired hydrolysis of endosomal PI(4,5)P2, thus highlighting the significance of sorting endosomes in the innate immune response. This notion was further corroborated by the results of RNAi-mediated knockdown of DPY-23, the μ subunit of the clathrin adaptor AP-2, which is anticipated to reduce endocytosis and sorting endosome formation (Pant et al., 2009), thus decreasing the survival rate of infected animals (Fig. S1C).

### UNC-16 serves as an effector of RAB-10 in sorting endosomes

RAB-10/Rab10 utilizes various effectors to regulate endocytic recycling (Zhang et al., 2022). To gain a better understanding of the role of RAB-10 in endosomal PI(4,5)P2 catabolism, we conducted a deficiency phenotype screening on previously reported effectors of RAB-10, including EHBP-1/Ehbp1, TBC-2/TBC1D2B, CNT-1/ACAPs, and HUM-2/myosin, as well as JIP-1/JIP1, KLC-1/kinesin, KLC-2/kinesin, and KLP-4/kinesin, which are involved in axial development (Chen et al., 2012; Deng et al., 2014; Liu and Grant, 2015; Shi et al., 2010, 2012). In this screening, our focus was particularly on the basolateral distribution of Tubby-PH^(R332H)^ and C1ab-PKD1^SC KI^ (Fig. S1D, top focal plane). The experiment showed no difference in the localization of both reporters, regardless of the absence of TBC-2, HUM-2, JIP-1, KLC-1, KLC-2, and KLP-4. Notably, despite a moderate accumulation of Tubby-PH^(R332H)^, we found that the pattern of C1ab-PKD1^SC KI^ was not altered in the absence of CNT-1 (Fig. S1E–Fʹ), indicating that endosomal synthesis of PI(4,5)P2 is not the primary factor impacting the occurrence of DAGs. In addition, we noticed that without EHBP-1, the distribution of Tubby-PH^(R332H)^ and C1ab-PKD1^SC KI^ was irregular, yet the labeling intensity of both reporters remained unchanged (Fig. S1E–Fʹ), indicating a change in endosomal architecture. These findings, taken together, point to an as-yet-undiscovered RAB-10-mediated process that governs the amount of endosomal PI(4,5)P2 and DAGs.

**Figure 2.**
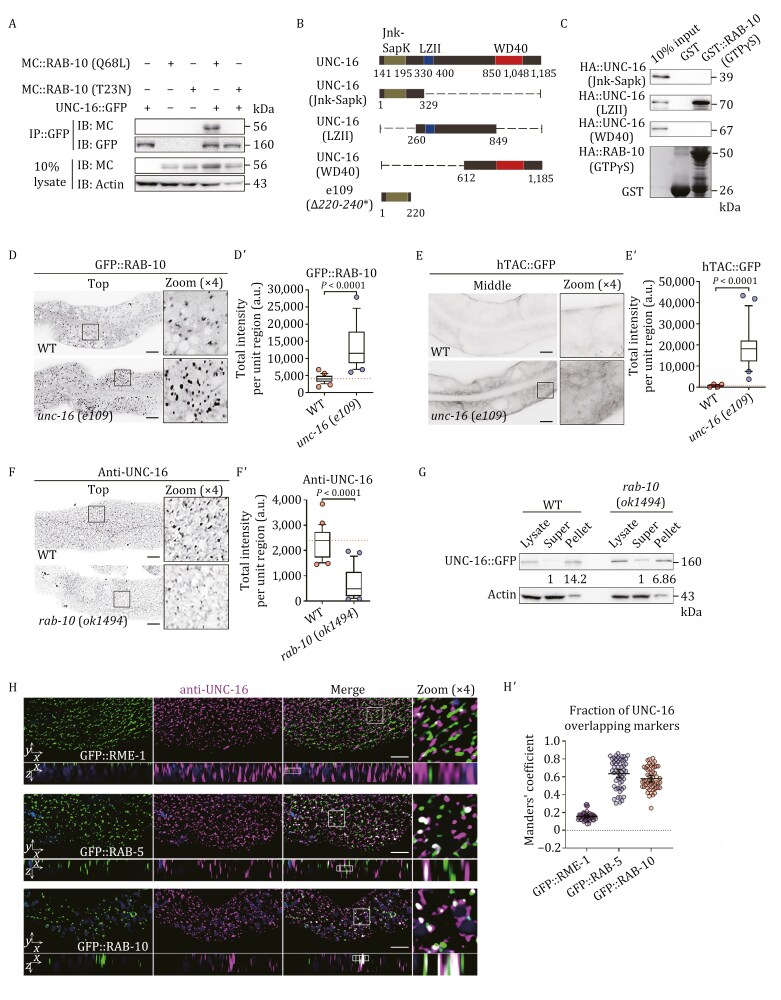
UNC-16 functions as an effector of RAB-10 in sorting endosomes. (A) Co-immunoprecipitation assays were performed using animals expressing epitope-tagged UNC-16, RAB-10(Q68L), and RAB-10(T23N). IB, immunoblot; IP, immunoprecipitation; MC, mCherry. (B) Domain architecture of UNC-16 and fragments containing Jnk-Sapk, LZII, WD40, and *e109*. Δ: deletion. (C) Western blot showing GST pull-down with *in vitro* translated HA-tagged UNC-16 domains. (D–Eʹ) Confocal images showing the localization of GFP::RAB-10 or hTAC::GFP in wild-type and *unc-16*(*e109*) animals. Top, focal plane near the basolateral membrane; middle, focal plane observing the deep cytosol. Box and whiskers plots (*n* = 24 regions): 10th–90th percentile; dots, outliers; red midline, median of wild-type; boundaries, quartiles. The *P*-values for the comparisons are indicated (two-tailed, unpaired *t*-test). Scale bars: 10 μm. (F and Fʹ) Confocal images showing the localization of UNC-16 in wild-type and *rab-10*(*ok1494*) animals. Box and whiskers plots (*n* = 24 regions): 10th–90th percentile; dots, outliers; midline, median of wild-type; boundaries, quartiles. The *P*-value for the comparison is indicated (two-tailed, unpaired *t*-test). Scale bars: 10 μm. (G) The membrane-to-cytosol ratio (P/S) of UNC-16 in wild-type and *rab-10*(*ok1494*) animals was measured. UNC-16 in the supernatants and pellets was analyzed by Western blot using an anti-GFP antibody. The loading control was blotted with the anti-β actin antibody. The average P/S ratio was calculated by normalizing the intensity of the supernatant or pellet with the corresponding β-actin, and the ratios are presented beneath the blots. (H and Hʹ) Confocal images showing the colocalization (upper panels: Z-axis focal plane; lower panels: Y-axis focal plane) between UNC-16 and GFP::RME-1, GFP::RAB-5, or GFP::RAB-10. The DAPI channel (blue color) indicates broad-spectrum intestinal autofluorescence. Mander’s coefficients were calculated using the Z-stack confocal slices. The data is the mean with 95% confidence intervals (*n* = 9 animals). Scale bars: 10 μm.

It has been indicated that JIP3/JIP4 functions as an effector of Arf6, regulating endosomal transport during cytokinesis (Montagnac et al., 2009). Recently, evidence has emerged that JIP3/JIP4 and Rab10 also have a physical interaction (Dumrongprechachan et al., 2022). Taking advantage of the sole homolog of JIP3 and JIP4 in *C*. *elegans*, UNC-16, we examined the interaction between UNC-16 and RAB-10. The results of the co-immunoprecipitation assay showed that UNC-16 only binds to the active RAB-10(Q68L), not to the inactive RAB-10(T23N) (Fig. 2A), suggesting that UNC-16 is a RAB-10 effector. To ascertain the primary binding region of UNC-16 for RAB-10, we divided UNC-16 into three fragments, each respectively containing a Jnk-Sapk, LZII (the second leucine zipper), and WD40 domain (Fig. 2B). An *in vitro* pull-down assay demonstrated that the fragment containing the LZII domain had an interaction with active RAB-10(GTPγS) (Fig. 2C).

To comprehend the functional relationship between RAB-10 and UNC-16 in intestinal cells, we utilized a null allele *unc-16*(*e109*), which has a deletion from the 220th to 260th amino acid residues, leading to an early termination codon (Figs. 2B and S2A). In *unc-16*(*e109*) animals, we noticed an intracellular buildup of GFP::RAB-10 on punctate structures (Fig. 2D and 2Dʹ); however, the membrane fractionation experiment did not show a significant increase in RAB-10’s association with endosomes (Fig. S2B). These findings indicate that the activity of RAB-10 is unaffected by UNC-16 deficiency, and the overaccumulation of RAB-10 is likely due to the aggregation of endosomal structures as a consequence of recycling malfunctions. Consistently, the accumulation of clathrin-independent recycling cargo hTAC (human IL-2 receptor α-chain) was observed in the intestinal cells of *unc-16*(*e109*) mutants (Fig. 2E and 2Eʹ). Likewise, GFP::RAB-5-labeled early/sorting endosomes accumulated in UNC-16-deficient animals, with the association of RAB-5 with the membrane remaining unaffected (Fig. S2C and S2D).

Next, we sought to determine the subcellular targeting mechanism of UNC-16. To accomplish this, we generated an antibody against UNC-16 and observed a reduction in punctate labeling of UNC-16 in *rab-10*(*ok1494*) mutants (Fig. 2F and 2Fʹ), suggesting that UNC-16 relies on recruitment by RAB-10 for its localization on endosomes. This finding was corroborated by the membrane fractionation assay, which employed the transgenic UNC-16::GFP, demonstrating that the lack of RAB-10 caused a diminished association of UNC-16 with the membrane (Fig. 2G). We then compared GFP::RME-1, a recycling endosome marker, with UNC-16 and found no significant overlap (Fig. 2H and 2Hʹ). Also, UNC-16 and ARF-6::GFP, another recycling endosome marker, showed very limited colocalization (Fig. S2E and S2Eʹ). In contrast, there was substantial colocalization between UNC-16 and early/sorting endosomal markers GFP::RAB-5 and GFP::RAB-10 (Fig. 2H and 2Hʹ), consistent with the notion that UNC-16 is a RAB-10 effector. It should be noted that UNC-16 is also present in RAB-7-labeled, ring-shaped late endosomal structures located in the deep cytosol (Fig. S2E and S2Eʹ), a result that is in line with the reported presence of JIP4 in lysosomes (Bonet-Ponce et al., 2020).

### UNC-16 deficiency causes a surge in endosomal PI(4,5)P2, a diminishment in DAGs, and inadequacies in the innate immune response

**Figure 3.**
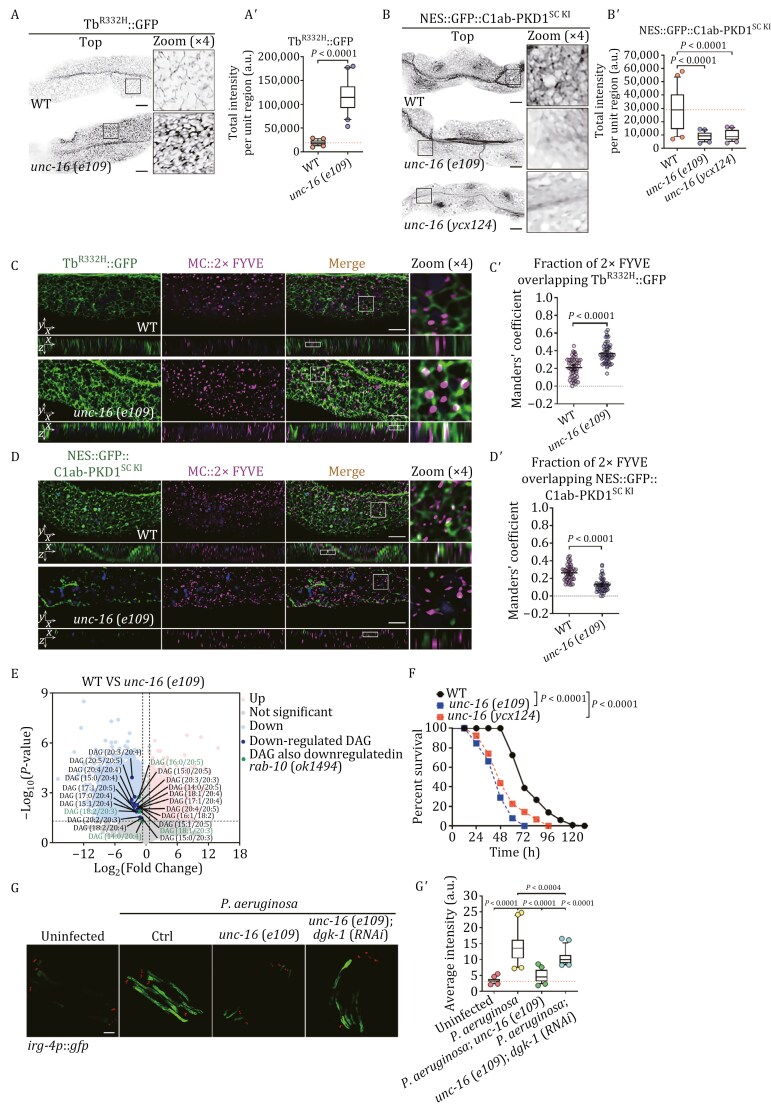
A deficiency in UNC-16 leads to an increase in endosomal PI(4,5)P2, a decrease in DAGs, and a lack of the innate immune response. (A–Bʹ) Confocal images showing the localization of Tubby-PH^(R332H)^::GFP and NES::GFP::C1ab-PKD1^SC KI^ in wild-type, *unc-16*(*e109*), or *ucn-16*(*ycx124*) animals. Box and whiskers plots (*n* = 24 regions): 10th–90th percentile; dots, outliers; midline, median of wild-type; boundaries, quartiles. The *P*-values for the comparison are indicated (two-tailed, unpaired *t*-test). Scale bars: 10 μm. (C–Dʹ) Confocal images showing the colocalization (upper panels: Z-axis focal plane; lower panels: Y-axis focal plane) between mCherry::2xFYVE and Tubby-PH^(R332H)^::GFP or NES::GFP::C1ab-PKD1^SC KI^ in *unc-16*(*e109*) animals. The DAPI channel (blue color) indicates broad-spectrum intestinal autofluorescence. Manders’ coefficients were calculated using the z-stack confocal slices. The data are the mean with 95% confidence intervals (*n* = 9 animals). The *P*-values for the comparisons are indicated (two-tailed, unpaired *t*-test). The scale bars are 10 μm. (E) A volcano plot illustrating the differential metabolite analysis between *unc-16*(*e109*) and wild-type animals is presented. Each point in the volcano plot represents a metabolite. The horizontal coordinates represent the fold change of the group comparing each substance (log_2_fold change), and the vertical coordinates represent the *P*-value of the *t*-test (−log_10_(*P*-value)). The raw data is filtered and categorized at *P* < 0.05, with a log_2_(fold change) >0.65 or <−0.65. Metabolites in the dataset that are significantly up-regulated are shown in pink, those that are significantly down-regulated are shown in blue, and those that are not significantly different are shown in gray. The DAGs that were determined to be significantly down-regulated are shown in dark blue, and those that were additionally observed to be downregulated in *rab-10*(*ok1494*) animals are depicted in green. See also Table S1. (F) Survival of wild-type, *unc-16*(*e109*, and *unc-16*(*ycx124*) animals exposed to *P*. *aeruginosa*. The *P*-values for the comparisons are indicated (log-rank test). Sample sizes and mean lifespan are shown in Table S2. (G and Gʹ) Confocal images of wild-type, *unc-16*(*e109*), and *unc-16*(*e109*);*dgk-1*(*RNAi*) animals expressing the transcriptional reporter *irg-4p*::*gfp* exposed to the indicated conditions. Box and whiskers plots (*n* = 25 animals): 10th–90th percentile; dots, outliers; midline, median of *wild-type* animals; boundaries, quartiles. The *P*-values for the comparisons are indicated (one-way ANOVA with Tukey’s multiple comparisons test). Scale bar, 100 µm.

Our findings point to UNC-16 as an effector of RAB-10 in sorting endosomes. Therefore, we are interested in investigating whether UNC-16 could be instrumental in maintaining PI(4,5)P2 homeostasis, as the absence of RAB-10 leads to a buildup of PI(4,5)P2 and a reduction in DAGs on sorting endosomes. First, we examined the intracellular distributions of Tubby-PH^(R332H)^ and C1ab-PKD1^SC KI^ in *unc-16*(*e109*) mutants. It was evident that the lack of UNC-16 led to a notable accumulation of Tubby-PH^(R332H)^ on the punctate and meshwork-like structures (Fig. 3A and 3Aʹ), while C1ab-PKD1^SC KI^ labeling on these structures was mostly absent (Fig. 3B and 3Bʹ). Subsequent analysis showed that structures exhibiting Tubby-PH^(R332H)^ accumulation were often co-labeled with 2xFYVE (Fig. 3C–Dʹ). In contrast, there was a notable decrease in C1ab-PKD1^SC KI^ on these 2xFYVE-labeled structures (Fig. 3C–Dʹ). These findings suggest that the deficiency of UNC-16 impacts the distribution of PI(4,5)P2 and DAGs in endosomes. Accordingly, we observed a significant decrease in endosomal labeling of Tubby-PH^(R332H)^ in intestinal cells overexpressing RAB-10(Q68L) and UNC-16, while an increase in labeling intensity was noted on these structures for C1ab-PKD1^SC KI^ (Fig. S3A–Bʹ). Next, we used liquid chromatography–tandem mass spectrometry (LC–MS/MS) to assess lipid levels (*n* = 3). To maximize the detection of DAG isomers, non-targeted lipidomics was employed, and the coverage of lipids was further increased by using both positive and negative ion polarity modes. We determined that 27,502 lipid molecules were present in total, of which 10,882 showed a significant alteration in abundance compared to wild-type animals (*P < *0.05 and log_2_(fold change) > 0.65 or <−0.65). Of these, 1,888 molecules were upregulated, and 8,994 were downregulated. Furthermore, a decrease in abundance of 22 DAG isomers in *unc-16*(*e109*) mutants was found, four of which were also observed in *rab-10*(*ok1494*) animals (16:0/20:5; 18:2/20:3; 18:1/20:3 and 14:0/20:4) (Fig. 3E, labeled in green). Consistently, upon exposure to *P*. *aeruginosa* infection, the survival rates of *unc-16*(*e109*) mutants drastically declined (Fig. 3F), whereas survival rates were heightened in animals with elevated UNC-16 expression (Fig. S3C). Also, overexpressing RAB-10(Q68L) led to a slight increase in survival rates, although without statistical significance (Fig. S3C).

To evaluate if UNC-16 primarily functions to resist pathogen invasion in intestinal epithelia, we also utilized the tissue-specific CRISPR/Cas9 technique to develop an intestine-specific knockout allele, *unc-16*(*ycx124*). We found that the C1ab-PKD1^SC KI^ labeling on the punctate and meshwork-like structures was significantly reduced, and the survival rate of *unc-16*(*ycx124*) animals was comparable to that of *unc-16*(*e109*) mutants (Figs. 3B, 3Bʹ and 3F), suggesting that the intestine is the primary tissue in which UNC-16 exerts regulatory activities in response to infection. We further evaluated the innate immune response elicited by *P*. *aeruginosa* infection. Remarkably, the upregulation of *irg-4p*::*gfp* was limited in *unc-16*(*e109*) mutants (Fig. 3G and 3Gʹ), which is in agreement with the phenotypes of RAB-10 deficiency. Corroborating this, further knockdown of DGK-1 in *unc-16*(*e109*) mutants partially revived the intensity of *irg-4p*::*gfp* (Fig. 3G and 3Gʹ). Taken together, our experiments suggest that UNC-16 and RAB-10 act in unison to manage the catabolism of endosomal PI(4,5)P2 and the associated innate immunity.

ARF-6/Arf6 has been identified as a facilitator of PI(4,5)P2 synthesis by recruiting PPK-1 (Chen et al., 2018; Donaldson, 2003; Shi et al., 2012). Additionally, it has been reported that JIP3/JIP4 is an interactor of Arf6 (Montagnac et al., 2009); thus, UNC-16 could also be involved in the regulation of PI(4,5)P2 synthesis. However, a substantial build-up of Tubby-PH^(R332H)^ was observed in endosomal structures in *unc-16*(*e109*) mutants, while the lack of ARF-6 led to a considerable decrease in Tubby-PH^(R332H)^ intensity (Fig. S3D and S3Dʹ). Notably, in the absence of UNC-16, the further loss of ARF-6 produced a phenotype with moderate intensity increase (Fig. S3D and S3Dʹ). As the sole homolog of the mammalian EHD, RME-1 is situated in the basolateral recycling endosome due to its affinity for PI(4,5)P2 (Pant et al., 2009; Shi et al., 2012). Upon examination of *unc-16*(*e109*) mutants, it was observed that RME-1 accumulated on the endosomal structures close to the plasma membrane, whereas the absence of ARF-6 led to a significant decrease in the labeling of RME-1 (Fig. S3E and S3Eʹ). In accordance with the pattern of Tubby-PH^(R332H)^, the labeling intensity of RME-1 in *unc-16*(*e109*);*arf-6*(*tm1447*) animals was more intense than in *arf-6*(*tm1447*) mutants (Fig. S3E and S3Eʹ). Altogether, these results suggest that UNC-16 is unlikely to act as an ARF-6 effector during PI(4,5)P2 synthesis, which is corroborated by the lack of notable colocalization between UNC-16 and ARF-6 (Fig. S2E and S2Eʹ).

Upon encountering a pathogen, *C*. *elegans* initiates a range of adaptive responses. One of these is the DAF-7/TGF-β neuroendocrine signaling pathway, which is required for the protective avoidance behavior towards *P*. *aeruginosa* (Meisel et al., 2014). Our findings, however, demonstrated that the absence of either RAB-10 or UNC-16 did not affect the animals’ avoidance response to *P*. *aeruginosa* (Fig. S3F), suggesting that the decreased survival of RAB-10- and UNC-16-deficient animals is not associated with deficits in their protective avoidance behavior.

### EGL-8/PLC-β is essential for the breakdown of endosomal PI(4,5)P2

**Figure 4.**
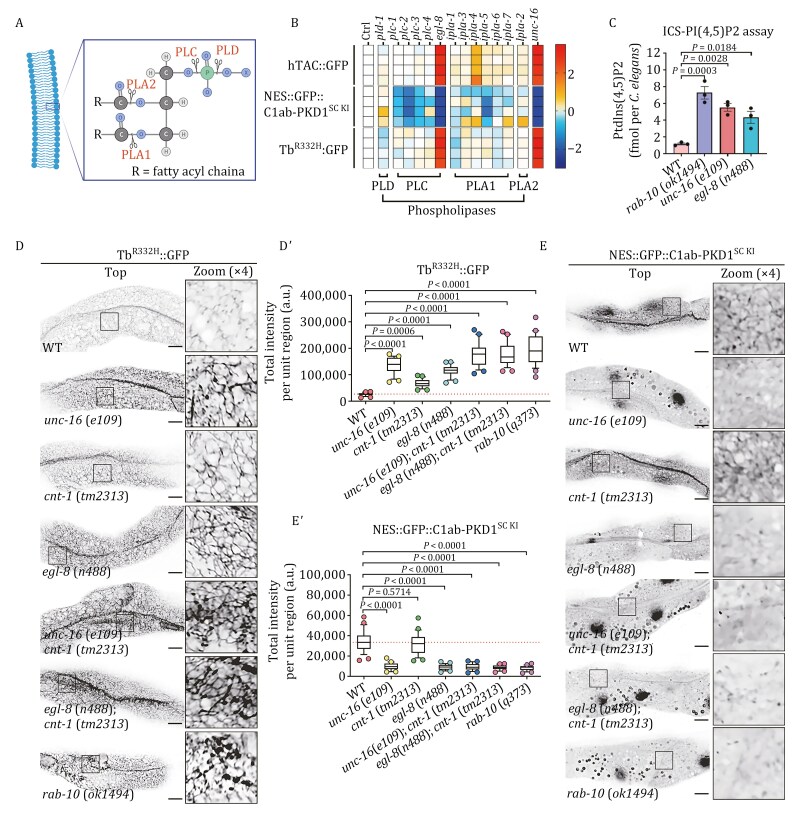
The EGL-8/PLC-β enzyme is required for the metabolic decomposition of endosomal PI(4,5)P2. (A) A diagram displays the cleavage sites of phospholipases on phospholipids. The locations for cutting are represented with scissors. O, oxygen; P, phosphorus; C, carbon; H, hydrogen. (B) The heatmap displays the levels of Tubby-PH^(R332H)^::GFP, hTAC::GFP, and NES::GFP::C1ab-PKD1^SC KI^ within cells after RNAi-mediated knockdown of 13 phospholipases, with unc-16(RNAi) as the phenotypical reference (*n* = 24, 4 regions × 6 animals). The relevant images are shown in Fig. S4. (C) An analysis was conducted using the Ion Chromatography System (ICS)-5000 to measure the levels of PI(4,5)P2 in wild-type, rab-10(ok1494), egl-8(n488), and unc-16(e109) animals. The averages of three replicates are displayed, with error bars showing the standard error of the mean (SEM). The *P*-values for the comparisons are shown (one-way ANOVA with Tukey’s multiple comparisons test). (D–Eʹ) Confocal images showing the localization of Tubby-PH^(R332H)^::GFP and NES::GFP::C1ab-PKD1^SC KI^ in wild-type, *unc-16*(*e109*), *cnt-1*(*tm2313*), *egl-8*(*n488*), *unc-16*(*e109*);*cnt-1*(*tm2313*), *egl-8*(*n488*);*cnt-1*(*tm2313*), and *rab-10*(*ok1494*) animals. Box and whiskers plots (*n* = 24 regions): 10th–90th percentile; dots, outliers; red midline, median of wild-type; boundaries, quartiles. The *P*-values for the comparison are indicated (one-way ANOVA with Dunnett’s multiple comparisons test). Scale bars: 10 μm.

Phospholipids can be metabolized into signaling molecules by phospholipases (Fig. 4A), which are classified into four types (PLA1/PLA2, PLB, PLC, and PLD) based on the site of cleavage (Kadamur and Ross, 2013; Wang and Tontonoz, 2019). Here, our inquiry suggested that the catabolism of endosomal PI(4,5)P2 is significant in regulating innate immunity. To substantiate the participation of phospholipase and pinpoint the exact enzyme, we conducted a screening by monitoring three phenotypes: hTAC::GFP accumulation in the cytosol as a sign of blocked recycling, Tubby-PH^(R332H)^ accumulation near the basolateral membrane as a manifestation of endosomal PI(4,5)P2 catabolic disorder, and decreased labeling of C1ab-PKD1^SC KI^ as an indication of DAG biogenesis deficiency. We screened phospholipases encoded in the *C*. *elegans* genome, including PLA1 (IPLA-1, IPLA-3, IPLA-4, IPLA-5, IPLA-6, IPLA-7), PLA2 (LPLA-2), PLC (PLC-1, PLC-2, PLC-3, PLC-4, EGL-8), and PLD (PLD-1), with the exception of IPLA-2/PLA1, as it does not express in the intestine (WormBase: WS290). Our results revealed that the phenotypes of cells lacking the PLC family member EGL-8/PLC-β are analogous to those of the *unc-16* mutants (Figs. 4B and S4). In contrast, the loss of PLC-1, PLC-2, and IPLA-5 led to a minor decrease in the labeling of C1ab-PKD1^SC KI^ (Fig. S4C and S4Cʹ), and the distribution and labeling of Tubby-PH^(R332H)^ and hTAC were not affected (Fig. S4A–Bʹ). Additionally, we observed that, in the absence of IPLA-4, there was a moderate accumulation of hTAC::GFP, yet the distribution of Tubby-PH^(R332H)^ and C1ab-PKD1^SC KI^ remained the same (Fig. S4A–Cʹ), suggesting that IPLA-4 could regulate recycling independently of phospholipid metabolism. To ascertain the alterations in PI(4,5)P2 levels more precisely, we utilized an ion chromatography system (ICS)-5000 to measure the PI(4,5)P2 of nematodes. The levels of PI(4,5)P2 were calculated using external calibration curves that were made with known concentrations of deacylated PI(4,5)P2 standard (diC16:0-PI(4,5)P2). Consistent with the imaging results, the levels of PI(4,5)P2 were substantially increased by 4–6 times in *rab-10*(*ok1494*) and *unc-16*(*e109*) mutants, and the absence of EGL-8 resulted in a 3-fold upregulation of PI(4,5)P2 (Fig. 4C). Taken together, our research suggests that EGL-8 is the phospholipase that catalyzes the hydrolysis of endosomal PI(4,5)P2, a process overseen by RAB-10 and UNC-16, ultimately leading to the production of DAGs.

RAB-10 exhibits its inhibitory effect on ARF-6 through the recruitment of CNT-1/ARF-6-GAP (Shi et al., 2012), inhibiting PPK-1-mediated PI(4,5)P2 synthesis. Interestingly, RAB-10-deficient cells showed a marked elevation in PI(4,5)P2 level, whereas the increase due to CNT-1 deficiency was comparatively minor (Shi et al., 2012). This finding raises the possibility that the elevation of endosomal PI(4,5)P2 due to RAB-10 deficiency is a synergistic consequence of augmented anabolism and diminished catabolism. We validated this proposition by showing that the accumulation of Tubby-PH^(R332H)^ in *unc-16*(*e109*);*cnt-1*(*tm2313*) double mutants was comparable to that in *egl-8*(*n488*);*cnt-1*(*tm2313*) animals (Fig. 4D and 4Dʹ), and the levels of Tubby-PH^(R332H)^ in these two types of double-mutants were analogous to that in RAB-10-deficient animals (Fig. 4D and 4Dʹ). Moreover, we noticed that the absence of UNC-16 or EGL-8 had a significant effect on the labeling of C1ab-PKD1^SC KI^ on endosomal structures, in contrast to no apparent influence of CNT-1 deficiency (Fig. 4E and 4Eʹ). Likewise, in *cnt-1*(*tm2313*) mutants, further loss of UNC-16 or EGL-8 resulted in a marked reduction in the presence of C1ab-PKD1^SC KI^ on these structures (Fig. 4E and 4Eʹ). Together, these data suggest that the anabolism of endosomal PI(4,5)P2 is not the rate-limiting step of DAGs biogenesis, which is corroborated by the fact that CNT-1 deficiency does not have a major impact on innate immunity even though it hinders PI(4,5)P2 synthesis in endosomes (Fig. 1B and 1Bʹ).

### UNC-16 directs the endosomal recruitment of EGL-8

**Figure 5.**
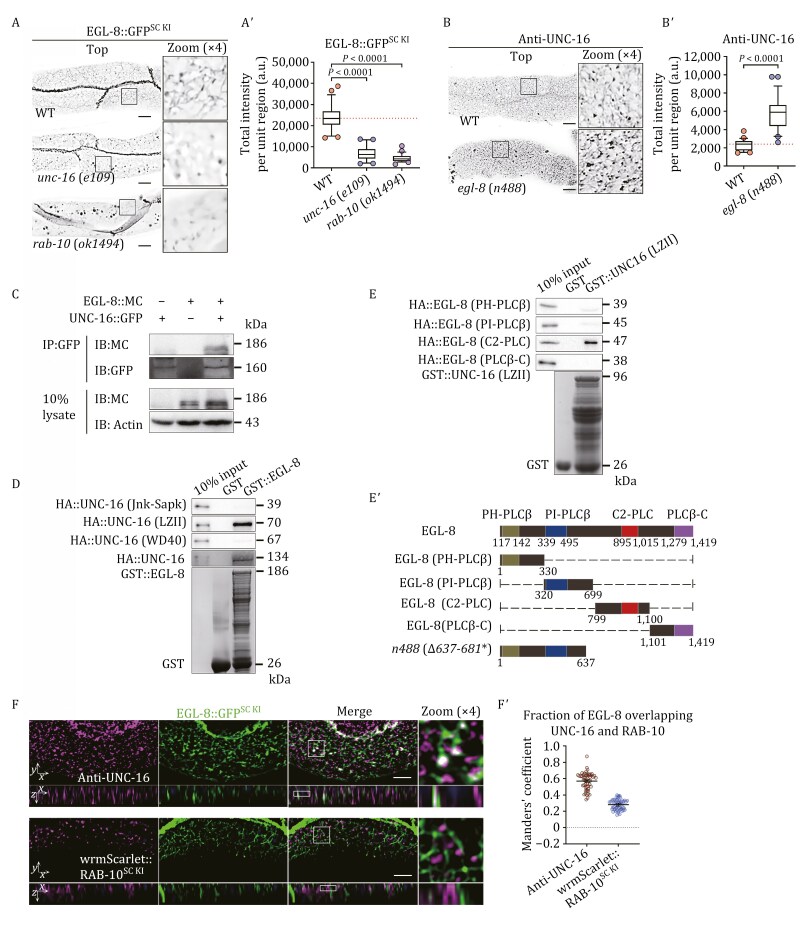
UNC-16 facilitates the endosomal recruitment of EGL-8. (A and Aʹ) Confocal images showing the localization of EGL-8::GFP^SC KI^ in wild-type, *unc-16(e109)*, and *rab-10*(*ok1494*) animals. Box and whiskers plots (*n* = 24 regions): 10th–90th percentile; dots, outliers; red midline, median of wild-type; boundaries, quartiles. The *P*-values for the comparison are indicated (one-way ANOVA with Tukey’s multiple comparisons test). Scale bars: 10 μm. (B and Bʹ) Confocal images showing the localization of endogenous UNC-16 in wild-type and *egl-8(n488)* animals. Box and whiskers plots (*n* = 24 regions): 10th–90th percentile; dots, outliers; midline, median of wild-type; boundaries, quartiles. The *P*-values for the comparison are indicated (two-tailed, unpaired *t*-test). Scale bars, 10 μm. (C) Co-immunoprecipitation assays were performed using animals expressing epitope-tagged EGL-8 and UNC-16. IB, immunoblot; IP, immunoprecipitation; MC, mCherry. (D) Western blot showing GST pull-down with *in vitro* translated HA-tagged UNC-16 domains. (E and Eʹ) Western blot showing GST pull-down with *in vitro* translated HA-tagged EGL-8 domains, and domain architecture of EGL-8 and fragments containing the PH-PLCβ (phospholipase C-β pleckstrin homology domain), PI-PLCβ (catalytic domain of metazoan phosphoinositide-specific phospholipase C-β), C2-PLC (C2 domain in phosphoinositide-specific phospholipases C), PLC-C (PLC-β C terminus), and *n488*. Δ: deletion. (F and Fʹ) Confocal images showing the colocalization (upper panels: Z-axis focal plane; lower panels: Y-axis focal plane) between EGL-8::GFP^SC KI^ and endogenous UNC-16 or wrmScarlet::RAB-10^SC KI^ in the intestinal cells. The DAPI channel (blue color) indicates broad-spectrum intestinal autofluorescence. Manders’ coefficients were calculated using the z-stack confocal slices. The data is mean with 95% confidence intervals (*n* = 9 animals). Scale bars, 10 μm.

The activity of PLC is often directed by G proteins, for instance, Gαq, which stimulates PLC-β (Waldo et al., 2010). Also, it has been observed that PLC-β is directly activated by small GTPases Rac1, Rac2, and Cdc42 (Illenberger et al., 1997, 1998). These reports lead us to hypothesize that EGL-8 might interact with RAB-10. We tested this by analyzing the distribution of EGL-8::GFP^SC KI^ and found that *rab-10*(*ok1494*) mutants had a reduced presence of EGL-8-labeled structures (Fig. 5A and 5Aʹ), suggesting that RAB-10 could recruit EGL-8 onto the endosomes. However, our co-immunoprecipitation assay failed to detect the interaction between EGL-8::mCherry and endogenous RAB-10 (Fig. S5A). To further examine this, we performed GST pull-down experiments using active RAB-10(GTPγS) and inactive RAB-10(GDP), which confirmed that there is no interaction between EGL-8 and RAB-10 (Fig. S5B), thereby ruling out the possibility of EGL-8 being directly recruited by RAB-10 onto endosomes for the purpose of PI(4,5)P2 hydrolysis.

InaD, a protein specific to *Drosophila* photoreceptors, has been proposed to act as a scaffold to interact with PLC-β via its fourth PDZ domain (Ranganathan and Ross, 1997). Intrigued by this, we sought to explore if UNC-16 could act as a scaffold protein to facilitate the endosomal recruitment of EGL-8. To do so, we examined the subcellular localization of EGL-8::GFP^SC KI^ in intestinal cells of *unc-16*(*e109*) mutant animals and also noticed a reduction in punctate labeling of EGL-8 (Fig. 5A and 5Aʹ). Conversely, in *egl-8*(*n488*) mutants, the endosomal residency of UNC-16 was augmented (Fig. 5B and 5Bʹ), suggesting that EGL-8 functions downstream of UNC-16, and UNC-16 is implicated in determining the endosomal localization of EGL-8. This speculation was corroborated by a co-immunoprecipitation assay, which revealed an interaction between EGL-8 and UNC-16 (Fig. 5C). Further pull-down experiments showed that UNC-16 was primarily bound to the C2 domain of EGL-8 via its LZII domain (Fig. 5D-Eʹ). Then, we compared the subcellular localization of EGL-8 with that of UNC-16 and RAB-10, and observed a notable overlap between EGL-8::GFP^SC KI^ and UNC-16 (Fig. 5F and 5Fʹ). However, the colocalization between EGL-8::GFP^SC KI^ and wrmScarlet::RAB-10^SC KI^ was not prominent (Fig. 5F and 5Fʹ), suggesting that the recruitment process between RAB-10, UNC-16, and EGL-8 probably occurs sequentially, rather than involving the assembly of a complex of all three proteins.

The PLC family consists of six subfamilies, including PLC-β, PLC-γ, PLC-δ, PLC-ε, PLC-ζ, and PLC-η (Katan and Cockcroft, 2020), with four of them being expressed in *C*. *elegans*: PLC-β (PLC-2 and EGL-8), PLC-γ (PLC-3), PLC-δ (PLC-4) and PLC-ε (PLC-1) (Yin et al., 2004). To further ascertain the functional specificity of EGL-8 in the hydrolysis of endosomal PI(4,5)P2, we examined the interaction between UNC-16 and PLC members. Our experiments demonstrated no interaction between UNC-16 and PLC-1, PLC-3, or PLC-4 (Fig. S5C). Of note, a feeble interaction was detected between UNC-16 and PLC-2 (Fig. S5C), which might be attributable to the fact that PLC-2/PLC-β and EGL-8 possess a similar structural composition. Further analysis of Tubby-PH^(R332H)^ in *unc-16*(*e109*) mutants revealed that overexpression of EGL-8 ameliorated the endosomal accumulation of PI(4,5)P2 in UNC-16-deficient cells, whereas overexpression of PLC-2, PLC-1, PLC-3, and PLC-4 had no such effect (Fig. S5D and S5Dʹ). Taken together, these results suggest that UNC-16 is responsible for recruiting EGL-8 to endosomes, and EGL-8 is particularly engaged in the catabolism of endosomal PI(4,5)P2.

### RAB-10, UNC-16, and EGL-8 work in tandem to activate the PMK-1/p38 MAPK innate immune pathway through the generation of DAGs

**Figure 6.**
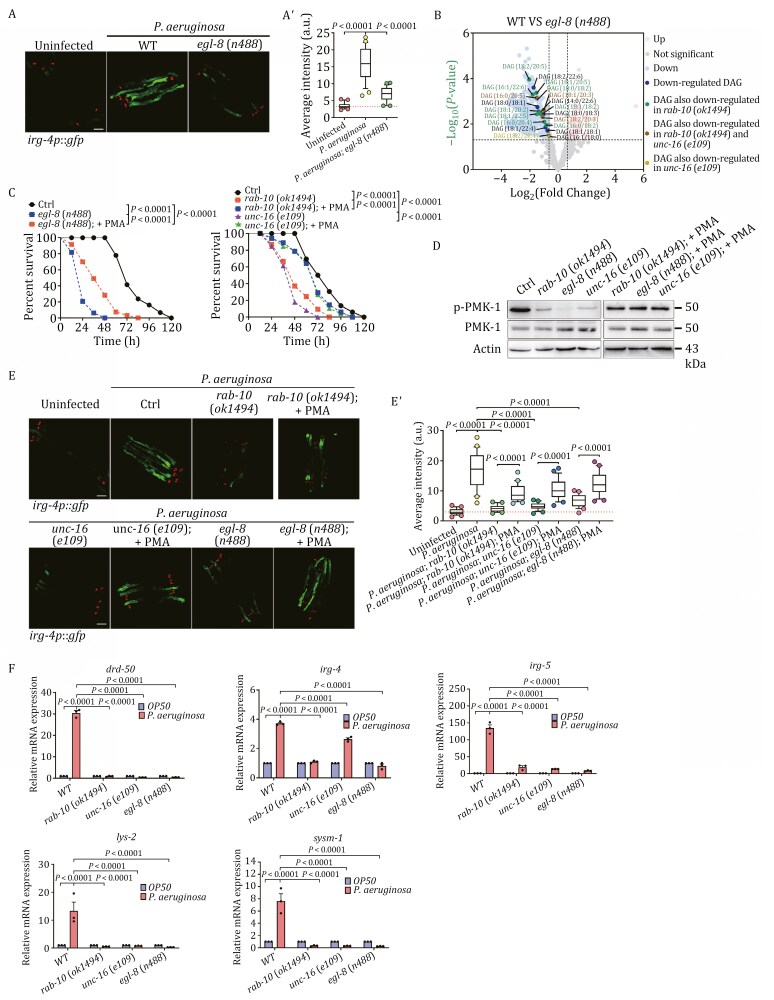
RAB-10, UNC-16, and EGL-8 collaborate to activate the PMK-1/p38 MAPK innate immune pathway through the production of DAGs. (A and A´) Confocal images of wild-type and *egl-8*(*n488*) animals expressing the transcriptional reporter *irg-4p*::*gfp* exposed to the indicated conditions. Box and whiskers plots (*n* = 25 animals): 10th–90th percentile; dots, outliers; red midline, median of *wild-type* animals; boundaries, quartiles. The *P*-values for the comparisons are indicated (one-way ANOVA with Tukey’s multiple comparisons test). Scale bar, 100 µm. (B) A volcano plot illustrating the differential metabolite analysis between *egl-8*(*n488*) and wild-type animals is presented. Each point in the volcano plot represents a metabolite. The horizontal coordinates represent the fold change of the group comparing each substance (log_2_(fold change)), and the vertical coordinates represent the *P*-value of the *t*-test (− log_10_(*P*-value)). The raw data is filtered and categorized at *P* < 0.05, with a log_2_(fold change) > 0.65 or <−0.65. Metabolites in the dataset that are significantly up-regulated are shown in pink, those that are significantly down-regulated are shown in blue, and those that are not significantly different are shown in gray. The DAGs that were determined to be significantly down-regulated are shown in dark blue, and those that were additionally observed to be downregulated in *rab-10*(*ok1494*) animals are depicted in green. Furthermore, those that were observed to be downregulated in both *rab-10*(*ok1494*) and *unc-16*(*e109*) animals are shown in brown, and those that were also observed to be downregulated in *unc-16*(*e109*) animals are depicted in yellow. See also Table S1. (C) Survival of wild-type and *egl-8*(*n488*), *rab-10*(*ok1494*), and *unc-16*(*e109*) animals was examined when exposed to *P*. *aeruginosa*, with and without PMA treatment. The *P*-values for the comparisons are indicated (log-rank test). Sample sizes and mean lifespan are shown in Table S2. (D) Immunoblots of animals with specified genotypes, with and without PMA treatment prior to exposure to *P*. *aeruginosa*. Antibodies that recognize the bisphosphorylated TGY motif of PMK-1 (phospho-p38 MAPK), total PMK-1 protein, and β actin were used for detection. (E and Eʹ) Confocal images of wild-type, *rab-10*(*ok1494*), *unc-16*(*e109*), and *egl-8*(*n488*) animals expressing the transcriptional reporter *irg-4p::gfp*, with and without PMA treatment, after exposure to *P*. *aeruginosa*. Box and whiskers plots (*n* = 25 animals): 10th–90th percentile; dots, outliers; midline, median of *wild-type* animals; boundaries, quartiles. The *P*-values for the comparisons are indicated (one-way ANOVA with Tukey’s multiple comparisons test). Scale bars, 100 µm. (F) qPCR analysis evaluating the expression of five PMK-1/p38 MAPK-dependent immunity genes (*drd-50*, *irg-4*, *irg-5*, *lys-2*, and *sysm-1*) in animals of the designated genotypes, both before and after exposure to *P*. *aeruginosa*. The data were the mean ± SEM of three replicates. The *P*-values for the comparisons are indicated (one-way ANOVA with Tukey’s multiple comparisons test).

EGL-8 is abundantly expressed in the nervous system and intestines, and its role in DAG-regulated biological process has been established in the context of synaptic transmission (Miller et al., 1999). To determine the implication of EGL-8 in innate immune response, we examined *irg-4p*::*gfp* expression in *egl-8*(*n488*) mutants after *P*. *aeruginosa* infection. Our findings showed that the upregulation of *irg-4p*::*gfp* was restricted in EGL-8-deficient animals (Fig. 6A and 6Aʹ), indicating that the intestinal innate immune response induced by *P*. *aeruginosa* requires EGL-8. Furthermore, we observed that overexpression of EGL-8 was beneficial to the survival of *rab-10*(*ok1494*) mutants during infection (Fig. S6A), as manifested by a marked rise in *irg-4p*::*gfp* (Fig. S6B and S6Bʹ), suggesting that EGL-8 plays a fundamental role in RAB-10-mediated innate immune response.

In *C*. *elegans*, DAGs act as the second messenger that activates the PMK-1/p38 MAPK innate immune pathway (Kawli et al., 2010); this is accomplished by activating TPA-1/PKC-δ, which then phosphorylates and activates DKF-2/PKD (Ren et al., 2009). Subsequently, DKF-2 phosphorylates TIR-1/SARM1 (Shirai and Saito, 2002; Ziegler et al., 2009). To substantiate the association between RAB-10, UNC-16, and EGL-8 in regulating innate immunity through DAGs, we performed a non-targeted lipidomics analysis (Fig. 6B). In comparison to wild-type animals, 499 lipid molecules were found to have differences in abundance in EGL-8-deficient animals (*P < *0.05 and log_2_(fold change) > 0.65 or <−0.65), of which 60 were upregulated, and 439 were downregulated. Notably, 12 of the 20 downregulated DAG isomers were observed in *rab-10*(*ok1494*) mutants and 4 in *unc-16*(*e109*) mutants. The levels of DAG (16:0/20:5), DAG (18:1/20:3), and DAG (18:2/20:3) were all decreased in animals deficient in RAB-10, UNC-16, and EGL-8 (Fig. 6B, labeled in brown). Phorbol-12 myristate-13 acetate (PMA/TPA) is widely used to imitate PKC regulation through the same mechanism as DAG (Damascena et al., 2022; Mosior and Newton, 1995). In particular, PMA demonstrates specificity by binding to the C1 domains within PKCs (Steinberg, 2008), thereby anchoring PKCs to membranes in their active conformations and activating them. To ascertain the specific activation of the p38 MAPK pathway by RAB-10, UNC-16, and EGL-8 through DAG signaling, we evaluated the effects of PMA on the survival of *rab-10*(*ok1494*), *unc-16*(*e109*), and *egl-8*(*n488*) mutant animals (Mosior and Newton, 1995). To prevent adverse effects on the immune system due to long-term exposure to PMA, we administered PMA (100 ng/mL) to the animals for six hours during the L4 larval stage (Kawli and Tan, 2008). Following treatment with PMA, the *rab-10*(*ok1494*), *unc-16*(*e109*), and *egl-8*(*n488*) mutants exhibited improved survival rates when exposed to *P*. *aeruginosa* (Fig. 6C), demonstrating the significant involvement of DAGs in the innate immunity mediated by RAB-10, UNC-16, and EGL-8. It should be noted that the *egl-8*(*n488*) animals showed a lower survival rate than *rab-10*(*ok1494*) and *unc-16*(*e109*) mutants (Fig. 6C), which can be attributed to their prominent vulnerability to environmental stresses.

Similar to mammalian p38 MAPKs, phosphorylation is the mechanism by which *C*. *elegans* PMK-1 is activated (Yuan et al., 2023). To directly gauge the activity of PMK-1, we opted to use the phospho-p38 MAPK (Thr180/Tyr182) antibody to measure the phosphorylation level of PMK-1 (Peterson et al., 2023). In *rab-10*(*ok1494*), *unc-16*(*e109*), and *egl-8*(*n488*) mutant animals, the phosphorylation levels of PMK-1 were found to be lower than those of wild-type animals (Fig. 6D). Nevertheless, upon treatment with PMA, significant increases in PMK-1 phosphorylation levels were observed (Fig. 6D). Similarly, PMA treatment resulted in an upregulation of *irg-4p*::*gfp* in *rab-10(ok1494)*, *unc-16*(*e109*), and *egl-8*(*n488*) mutants infected with *P*. *aeruginosa* (Fig. 6E and 6Eʹ). The PMK-1/p38 MAPK innate immune pathway is a major contributor to innate immunity in *C*. *elegans*, particularly in the intestinal cells (Kim et al., 2002; Peterson et al., 2022; Shivers et al., 2009), and its effector genes, including *drd-50*, *irg-4*, *irg-5*, *lys-2*, and *sysm-1*, have been the focus of multiple studies (Nandakumar and Tan, 2008; Peterson et al., 2019, 2023; Soltanmohammadi et al., 2022). We also assessed the transcriptional response of these genes to *P*. *aeruginosa* exposure. Notably, the mRNA level of *drd-50* increased by nearly 30-fold (Fig. 6F), and the mRNA levels of *irg-4*, *irg-5*, *lys-2*, and *sysm-1* rose by approximately 4-, 120-, 10-, and 7-fold, respectively (Fig. 6F). Moreover, in the absence of RAB-10, UNC-16, and EGL-8, the expression of these genes was significantly diminished (Fig. 6F). Hence, we concluded that the RAB-10-UNC-16-EGL-8 module is accountable for regulating the activity of the PMK-1/p38 MAPK pathway and its ensuing innate immune efficacy.

### UNC-16 dimerization is crucial for its interaction with RAB-10 and EGL-8

**Figure 7.**
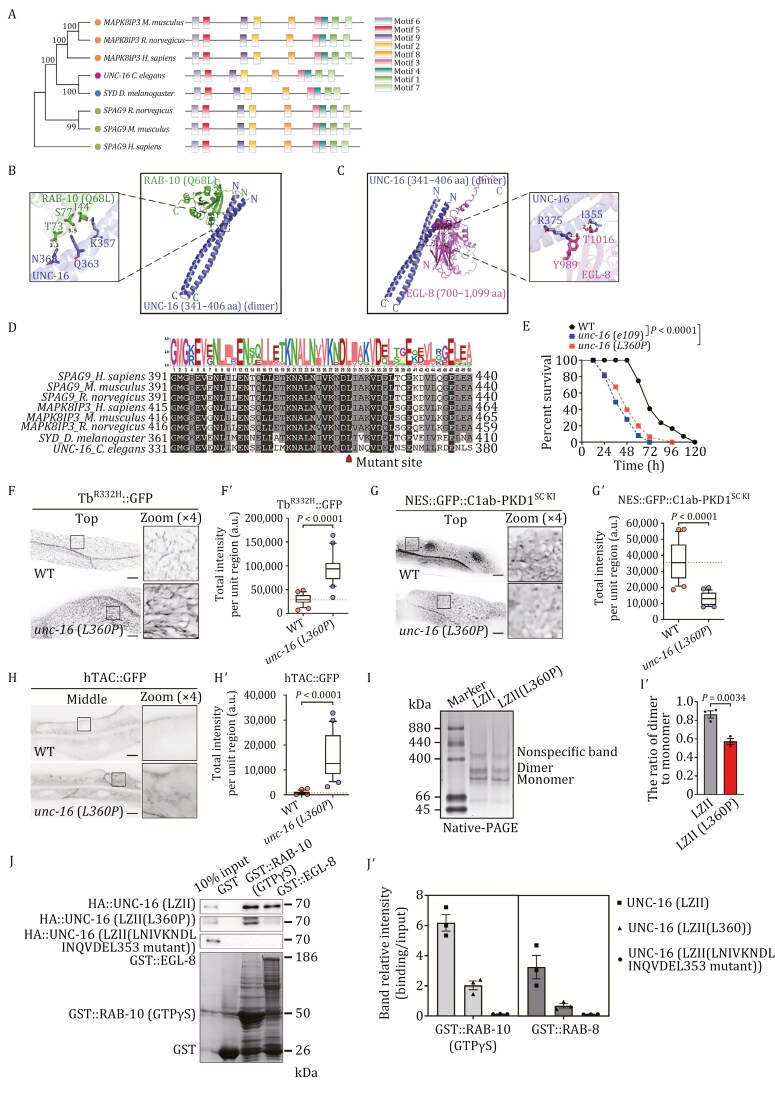
UNC-16 dimerization through motif 9 is essential for its interaction with RAB-10 and EGL-8. (A) A phylogenetic comparison of UNC-16 and its homologs JIP3/MAPK8IP3, JIP4/SPAG9, and SYD/Sunday Driver from multiple species using MUltiple Sequence Comparison by Log-Expectation (MUSCLE) and the neighbor-joining method. Bootstrap values are indicated at nodes, and protein motifs were obtained from full-length sequences using the MEME tool, with rectangles of varying colors representing the different conserved motifs, scaled to show the length of each motif. (B and C) AlphaFold2-Multimer was employed to predict the structure of complexes comprising the dimeric UNC-16-LZII core region (341–406 aa) and either RAB-10 (Q68L) or EGL-8 (700–1,099 aa). Magnified views display the amino acid residues participating in the interaction. (D) The MEME Suite was used to analyze the sequence conservation of UNC-16 (331–380 aa) and its homologous regions from multiple species. The height of the stacks symbolizes the sequence conservation at that position, and the height of the letter within each stack symbolizes the relative frequency of the corresponding amino acid. The red arrow indicates the mutation site of *unc-16*(*L360P*) mutant. (E) Survival of wild-type, *unc-16*(*e109*), and *unc-16*(*L360P*) animals were examined when exposed to *P. aeruginosa.* The *P*-values for the comparisons are indicated (log-rank test). Sample sizes and mean lifespan are shown in Table S2. (F–Hʹ) Confocal images showing the localization of Tubby-PH^(R332H)^::GFP, NES::GFP::C1ab-PKD1^SC KI^, and hTAC::GFP in wild-type and *unc-16*(*L360P*) animals. Box and whiskers plots (*n* = 24 regions): 10th–90th percentile; dots, outliers; midline, median of wild-type; boundaries, quartiles. The *P*-values for the comparison are indicated (two-tailed, unpaired *t*-test). Scale bars: 10 μm. (I and Iʹ) Native-PAGE of purified UNC-16-LZII domain and its L360P variant (260–849 aa) was performed. The histogram represents data from three independent experiments. The mean ± SEM is shown. The *P*-value for the comparison is indicated (two-tailed, paired *t*-test). (J and Jʹ) Western blot showing GST pull-down with *in vitro* translated HA-tagged UNC-16(LZII), UNC-16(LZII)(L360P), and UNC-16(LZII) with all seven amino acids surrounding L360P mutated to alanine. The histogram illustrates the results of three independent replicates.

UNC-16, alongside its homologs JIP3/MAPK8IP3, JIP4/SPAG9, and Sunday Driver, have been established as scaffold proteins in microtubule-assisted transports (Byrd et al., 2001; Cavalli et al., 2005; Snead and Gowrishankar, 2022). To gain an in-depth understanding of the evolution of UNC-16 homologs, we resorted to the neighbor-joining method to infer their evolutionary trajectory (Saitou and Nei, 1987). Poisson correction was utilized to compute the evolutionary distances, which are measured in the number of amino acid substitutions per site (Fig. 7A). Our results indicated that UNC-16 and SYD are more closely related to JIP3/MAPK8IP3 than JIP4/SPAG9 (Fig. 7A). Then, MEME tools were employed to identify conserved motifs and TBtools was used to create data visualizations (Bailey and Elkan, 1994; Chen et al., 2020), which revealed that UNC-16 and its homologs are highly conserved, each consisting of nine motifs (Fig. 7A).

Structural analysis has shown that the second leucine zipper (LZII) domain of JIP4 interacts with Arf6 to create a heterotetramer with an Arf6-(JIP4)(2)-Arf6 configuration (Isabet et al., 2009). Also, JIP3-LZII was identified as the region that binds to the tetratricopeptide repeat domain of the kinesin-1 light chain (Cockburn et al., 2018). Captivated by these reports, we utilized AlphaFold2-Multimer to explore the functional intricacies of the UNC-16 LZII domain (330–400 aa) by predicting the structures of complexes formed by the UNC-16-LZII core region (341–406 aa) and either RAB-10 or EGL-8 C-terminal fragment (700–1,099 aa) (Jumper et al., 2021). Our inquiry showed that UNC-16-LZII assembles into homodimers via its coiled-coil regions and binds to RAB-10 through the residues K357, Q363, and N368 (Fig. 7B). In addition, the homodimer interacts with EGL-8 through I355 and R375 in the same region (Fig. 7C). However, we noticed that RAB-10 and EGL-8 interact with the UNC-16-LZII homodimer in distinct manners, attaching to opposite sides of the coiled-coil structure (Fig. 7B and 7C). Moreover, it is noteworthy that the residues predicted to be involved in the interaction between LZII and either RAB-10 or EGL-8 are mainly situated in motif 9 (331–380 aa) (Fig. 7D), highlighting the significance of motif 9 in UNC-16 dimerization and its relevant activities.

Exon sequencing of individuals with neurodevelopmental disorders has uncovered two mutations (Leu444 and Glu461) in the MAPK8IP3 LZII domain, located in motif 9 (Platzer et al., 2019). Analysis of the structure indicated that these mutations could interfere with leucine zipper homodimeric interactions (Platzer et al., 2019). In addition, patients with MAPK8IP3 mutations often display a weakened immune system (Firth et al., 2009). This leads to the inquiry of whether a mutation in the analogous site of UNC-16 could have a detrimental effect on its functionality. To this end, we used CRISPR/Cas9 genome editing to introduce the L444P mutation of MAPK8IP3 into the corresponding site (L360P) in UNC-16 (Fig. 7D). Of note, the survival rate of *unc-16*(*L360P*) mutant animals was reduced when exposed to *P*. *aeruginosa* (Fig. 7E). This mutation also resulted in an endosomal accumulation of Tubby-PH^(R332H)^ and a decrease in the labeling of C1ab-PKD1^SC KI^ (Fig. 7F–Gʹ). Moreover, recycling cargo hTAC::GFP was observed to accumulate in the deep cytosol of *unc-16*(*L360P*) mutants (Fig. 7H and 7Hʹ). These findings were further supported by the observation that the accumulations of Tubby-PH^(R332H)^ and hTAC::GFP in *unc-16*(*e109*) animals were alleviated by overexpression of UNC-16, but not by the excessive presence of UNC-16(L360P) (Fig. S7A–Bʹ). Then, we tested the effect of the L360P mutation on the formation of UNC-16 homodimer by using purified fragments bearing the LZII domain or its L360P variant (260–849 aa). Our results, observed from native-PAGE, showed a decrease in the formation of dimers for the L360P variant (Fig. 7I and 7Iʹ). Furthermore, the introduction of the L360P mutation reduced the interaction of UNC-16-LZII with EGL-8 and RAB-10(GTPγS). When L360 and its seven neighboring amino acids were all substituted with alanine, the binding of the LZII domain to EGL-8 and RAB-10(GTPγS) was almost abolished (Fig. 7J and 7Jʹ). Consistently, overexpression of UNC-16(L360P) failed to restore the subcellular labeling of EGL-8 in UNC-16-deficient animals (Fig. S7C and S7Cʹ). Collectively, these results suggest that the formation of the UNC-16 homodimer, facilitated by the LZII domain, is a prerequisite for the RAB-10-controlled endosomal recruitment of EGL-8, thus promoting the hydrolysis of PI(4,5)P2 and the production of DAGs.

### *P*. *aeruginosa* infection induces an increase in RAB-10 activity, and this is likely attributed to the amplified expression of LET-413

**Figure 8.**
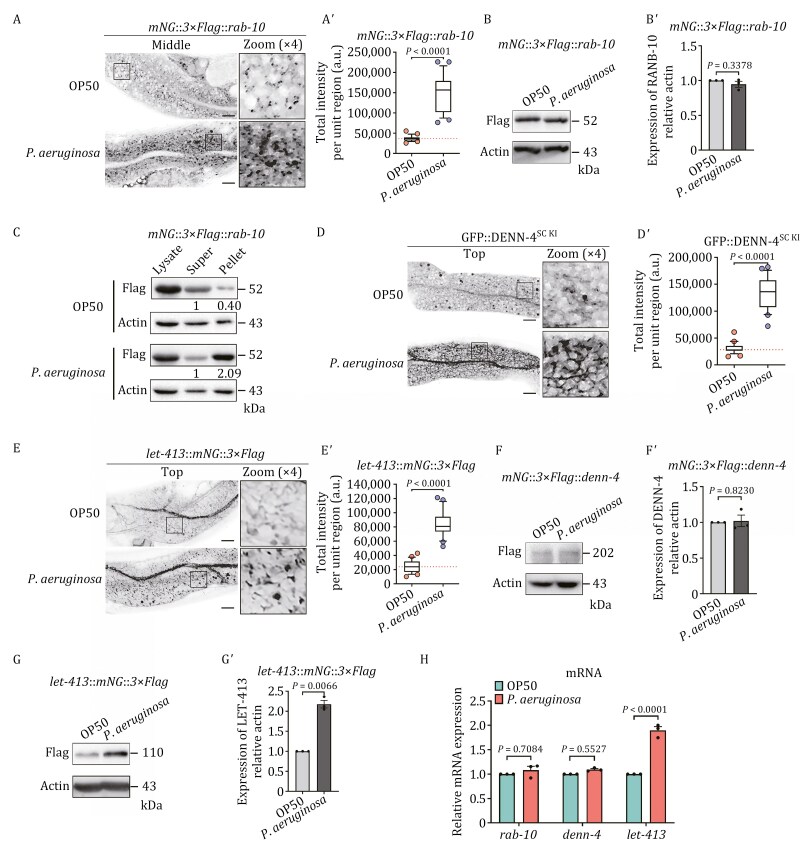
*P. aeruginosa* infection leads to an increase in RAB-10 activity, which is likely attributed to the enhanced expression of LET-413. (A and Aʹ) Confocal images showing the localization of endogenous RAB-10 in the absence or presence of *P*. *aeruginosa*. Box and whiskers plots (*n* = 24 regions): 10th–90th percentile; dots, outliers; red midline, median of wild-type; boundaries, quartiles. The *P*-value for the comparison is indicated (two-tailed, unpaired *t*-test). Scale bars, 10 μm. (B and Bʹ) Western blot showing the endogenous levels of RAB-10. The relative intensity of RAB-10 was quantified relative to actin. The data were the mean ± SEM of three replicates. The *P*-value for the comparison is indicated (two-tailed, paired *t*-test). (C) The membrane-to-cytosol ratio (P/S) of endogenous RAB-10 in animals exposed to OP50 or *P*. *aeruginosa* was measured. RAB-10 in the supernatants and pellets was analyzed by Western blot using an anti-Flag antibody. The loading control was blotted with the anti-β actin antibody. The average P/S ratio was calculated by normalizing the intensity of the supernatant or pellet with the corresponding β-actin, and the ratios are presented beneath the blots. (D–Eʹ) Confocal images showing the localization of GFP::DENN-4^SC KI^ and endogenous LET-413 in the absence or presence of *P*. *aeruginosa*. Box and whiskers plots (*n* = 24 regions): 10th–90th percentile; dots, outliers; midline, median of wild-type; boundaries, quartiles. The *P*-values for the comparison are indicated (two-tailed, unpaired *t*-test). Scale bars, 10 μm. (F–Gʹ) Western blot showing the endogenous levels of DENN-4 and LET-413. The relative intensity of RAB-10 was quantified relative to actin. The data were the mean ± SEM of three replicates. The *P*-values for the comparison are indicated (two-tailed, paired *t*-test). (H) qPCR analysis evaluating the expression of *rab-10*, *denn-4*, and *let-413* before and after exposure to *P*. *aeruginosa*. The data were the mean ± SEM of three replicates. The *P*-values for the comparison are indicated (one-way ANOVA with Bonferroni’s multiple comparisons test).

Thus far, our research has established that active RAB-10 brings UNC-16 to the endosome, thereby enabling the recruitment of EGL-8 to trigger the PMK-1/p38 MAPK immune response. To further scrutinize how nematodes elevate the activity of this pathway in response to infection, we examined the distribution of endogenous RAB-10 during infection with *P*. *aeruginosa* and discovered an accumulation of RAB-10 in intestinal cells (Fig. 8A and 8Aʹ), which could be due to an enhanced relocation of RAB-10 from the cytoplasm to endosomes or an increase in the expression of RAB-10. However, the protein level of RAB-10 did not appear to be significantly affected (Fig. 8B and 8Bʹ), yet the membrane fragmentation assay indicated that the association of RAB-10 with the membrane was increased in animals infected with *P*. *aeruginosa* (Fig. 8C). Given that only active Rabs can effectively localize to endosomes (Grosshans et al., 2006), our findings suggest that *P*. *aeruginosa* infection could enhance RAB-10 activity.

DENN-4/DENND4 acts as a GEF (guanine nucleotide exchange factor) for RAB-10, and its interaction with LET-413 is necessary for the activation of RAB-10 (Liu et al., 2018; Sano et al., 2011; Yoshimura et al., 2010). To gain insight into how *P*. *aeruginosa* infection promotes RAB-10 activity, we sought to analyze the subcellular localization of DENN-4 and LET-413 following infection. We found that both GFP::DENN-4^SC KI^ and endogenous LET-413 (LET-413::mNG::3xFlag) aggregated on punctate and meshwork-like endosomal structures (Fig. 8D–Eʹ). Additionally, the expression of LET-413 was significantly increased, while the expression of DENN-4 remained unchanged (Fig. 8F–Gʹ). A qPCR assay further confirmed that the mRNA level of LET-413 was elevated in infected animals (Fig. 8H). It has been proposed that TBC-11/AS160/TBC1D4 acts as a GAP (GTPase activating protein) for RAB-10 (Sano et al., 2007, 2011); however, the mRNA level of TBC-11 was not noticeably affected by infection (Fig. S7D). Taken together, our findings point to the fact that RAB-10 activity is heightened during *P*. *aeruginosa* infection, and this is likely due to the increased expression of LET-413.

### NHR-25, a nuclear receptor, is required to increase LET-413 expression in the context of *P*. *aeruginosa* infection

**Figure 9.**
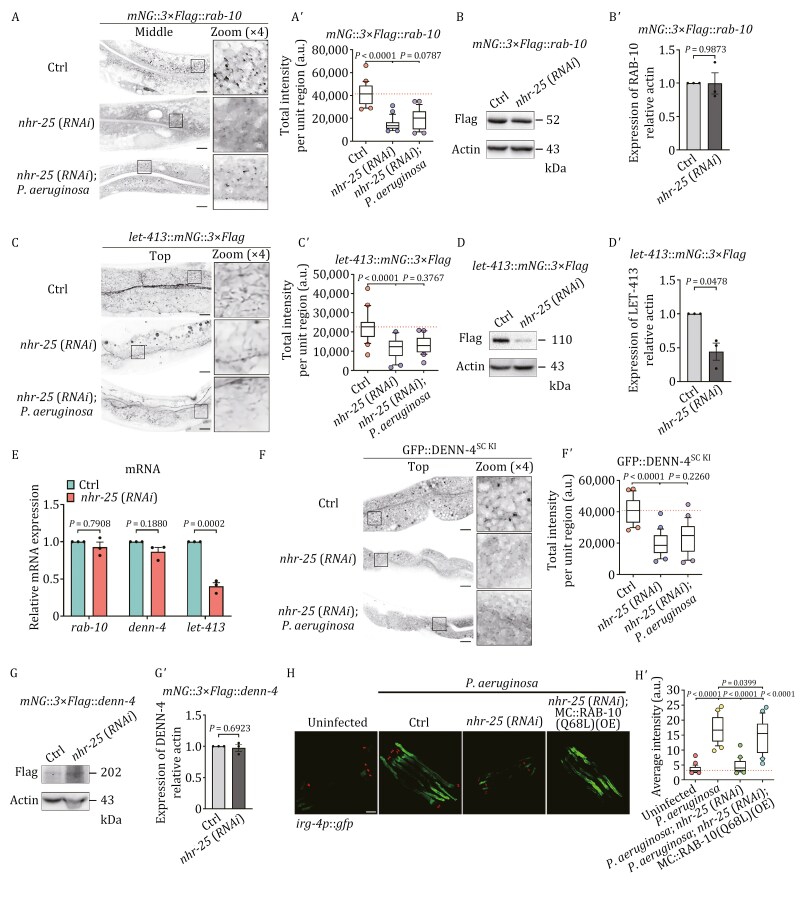
NHR-25, a nuclear receptor, is required for the enhanced expression of LET-413 in the context of *P. aeruginosa* infection. (A and Aʹ) Confocal images showing the localization of endogenous RAB-10 in *nhr-25(RNAi)* animals, both when *P*. *aeruginosa* was present or absent. Box and whiskers plots (*n* = 24 regions): 10th–90th percentile; dots, outliers; red midline, median of wild-type; boundaries, quartiles. The *P*-value for the comparison is indicated (one-way ANOVA with Dunnett’s multiple comparisons test). Scale bars, 10 μm. (B and Bʹ) Western blot showing the endogenous levels of RAB-10. The relative intensity of RAB-10 was quantified relative to actin. The data were the mean ± SEM of three replicates. The *P*-value for the comparison is indicated (two-tailed, paired *t*-test). (C and Cʹ) Confocal images show the localization of endogenous LET-413 in *nhr-25(RNAi)* animals, both when *P*. *aeruginosa* was present or absent. Box and whiskers plots (*n* = 24 regions): 10th–90th percentile; dots, outliers; red midline, median of wild-type; boundaries, quartiles. The *P*-value for the comparison is indicated (one-way ANOVA with Dunnett’s multiple comparisons test). Scale bars, 10 μm. (D and Dʹ) Western blot showing the endogenous levels of LET-413. The relative intensity of LET-413 was quantified relative to actin. The data were the mean ± SEM of three replicates. The *P*-value for the comparison is indicated (two-tailed, paired *t*-test). (E) qPCR analysis evaluating the expression of *rab-10*, *denn-4*, and *let-413* in wild-type and *nhr-25*(*RNAi*) animals. The data were the mean ± SEM of three replicates. The *P*-values for the comparison are indicated (one-way ANOVA with Bonferroni’s multiple comparisons test). (F and Fʹ) Confocal images show the localization of endogenous DENN-4 in *nhr-25(RNAi)* animals, both when *P*. *aeruginosa* was present or absent. Box and whiskers plots (*n* = 24 regions): 10th–90th percentile; dots, outliers; midline, median of wild-type; boundaries, quartiles. The *P*-value for the comparison is indicated (one-way ANOVA with Dunnett’s multiple comparisons test). Scale bars, 10 μm. (G and Gʹ) Western blots show the endogenous levels of DENN-4. The relative intensity of DENN-4 was quantified relative to actin. The data were the mean ± SEM of three replicates. The *P*-value for the comparison is indicated (two-tailed, paired *t*-test). (H and Hʹ) Confocal images of wild-type, *nhr-25*(*RNAi*), and *nhr-25*(*RNAi*);mCherry::RAB-10(Q68L) (*OE*) animals expressing the transcriptional reporter *irg-4p*::*gfp* after exposure to *P*. *aeruginosa*. Box and whiskers plots (*n* = 25 animals): 10th–90th percentile; dots, outliers; red midline, median of *wild-type* animals; boundaries, quartiles. The *P*-values for the comparisons are indicated (one-way ANOVA with Tukey’s multiple comparisons test). Scale bar, 100 µm.

An analysis of 271 nuclear hormone receptors in *C*. *elegans* revealed that the suppression of *nhr-25* expression led to a decrease in *irg-4p*::*gfp* expression (Peterson et al., 2023), suggesting that NHR-25/NR5A1/2 is a nuclear receptor involved in perceiving pathogens. Furthermore, as a nuclear receptor family transcription factor, its binding site was identified in the *let-413* promoter region (Araya et al., 2014; Riga et al., 2021). To evaluate the necessity of NHR-25 for the upregulation of LET-413 and subsequent rise in RAB-10 activity in response to *P*. *aeruginosa* infection, we first examined the distribution of endogenous RAB-10 in *nhr-25*(*RNAi*) animals. Our results indicated that the labeling of RAB-10-positive punctate structures decreased and failed to regain their levels after infection (Fig. 9A and 9Aʹ), yet the protein and mRNA levels of RAB-10 remained unaltered (Fig. 9B, 9Bʹ, and 9E). Notably, in NHR-25-deficient cells, endogenous LET-413 was largely absent from the punctate and meshwork-like structures, regardless of the presence or absence of *P*. *aeruginosa* (Fig. 9C and 9Cʹ). Moreover, there was a noticeable decline in LET-413 expression in *nhr-25*(*RNAi*) animals (Fig. 9D, 9Dʹ, and 9E). Previous studies have demonstrated that the depletion of LET-413 affects the labeling of DENN-4 on membrane tubules and puncta (Liu et al., 2018). Accordingly, we observed that NHR-25-deficient cells had a marked decrease in DENN-4-labeled structures irrespective of infection (Fig. 9F and 9Fʹ), while the level of DENN-4 expression stayed unchanged (Fig. 9G, 9Gʹ, and 9E). Together, these results suggest that NHR-25 is implicated in enhancing LET-413 expression and, consequently, activating RAB-10. This is further evidenced by the reduced expression of *irg-4p*::*gfp* in the absence of NHR-25 when compared to wild-type animals (Fig. 9H and 9Hʹ). In addition, the intensity of *irg-4p*::*gfp* was restored in *nhr-25(RNAi)* animals upon overexpression of a constitutively active RAB-10(Q68L) (Fig. 9H and 9Hʹ).

To gain further insight into NHR-25’s effect on the RAB-10-mediated endosomal PI(4,5)P2 hydrolysis, we studied the distribution and expression of UNC-16 and EGL-8 in animals affected by *P*. *aeruginosa*. Our results showed that the UNC-16 labeling on endosomal structures increased, but the protein level remained unchanged (Fig. S8A–Bʹ). Similarly, EGL-8 accumulated on punctate structures without any alteration in protein levels (Fig. S8C–Dʹ). Quantitative PCR analysis corroborated these results (Fig. S8E). Collectively, these data suggest that NHR-25 is solely accountable for the increased expression of LET-413, without impacting the levels of UNC-16 and EGL-8.

It has recently been reported that NHR-86/HNF4, a nuclear hormone receptor, is a phenazine-1-carboxamide (PCN) sensor for *C. elegans*, activating a transcriptional program in the intestinal epithelium to safeguard against *P. aeruginosa* infection (Peterson et al., 2023). We are curious to find out if NHR-86 also has any effect on the subcellular location of RAB-10. Following the RNAi-mediated knockdown of NHR-86, the presence of RAB-10 on the punctate structure was not significantly altered (Fig. S8F and S8Fʹ). Likewise, in the presence of *P*. *aeruginosa*, the labeling of RAB-10 was still increased in *nhr-86*(*RNAi*) animals (Fig. S8F and S8Fʹ), while the protein level of RAB-10 was maintained (Fig. S8G), suggesting that NHR-86-mediated PCN perception is unlikely to be involved in the *P*. *aeruginosa*-induced upregulation of LET-413.

G protein-coupled receptors (GPCRs) have been extensively studied for their role in the innate immunity of *C*. *elegans*, with FSHR-1 being particularly noteworthy (Powell et al., 2009). This receptor is an essential component of the intestinal immune system, sending signals in tandem with the p38 MAPK pathway to regulate the transcription of specific antimicrobial effectors (Powell et al., 2009). Additionally, research has suggested that the G protein α q subunit (EGL-30) can activate EGL-8 to produce DAGs, which can then influence neural secretions (Lackner et al., 1999). Subsequent investigations demonstrated that both EGL-30 and EGL-8 are necessary for the proper functioning of the immune system (Kawli et al., 2010). To further explore this, experiments were conducted to assess whether FSHR-1 and EGL-30 are involved in the concurrent regulation of the distribution of RAB-10. However, the absence of FSHR-1 or EGL-30 had no effect on the punctate pattern of RAB-10, nor did it alter the intensity of the labeling (Fig. S8F, S8Fʹ, and S8G). The elevation of RAB-10 labeling caused by *P*. *aeruginosa* infection was also unaffected by the lack of FSHR-1 or EGL-30 (Fig. S8F and S8Fʹ). These findings point to the possibility that the immune responses regulated by FSHR-1 and EGL-30 are not reliant on PI(4,5)P2 catabolism in sorting endosomes, a process managed by RAB-10.

It has been demonstrated that the δ-opioid receptor binds to Rab10, as determined by mass spectrometry (Degrandmaison et al., 2020). The *C*. *elegans* genome encodes four opioid receptors: NPR-17, NPR-23, NPR-30, and NPR-29, with NPR-17 and NPR-30 expressed in intestinal cells (Blazie et al., 2017; Cheong et al., 2015). While it is plausible that opioid receptors may play a role in activating RAB-10 upon detection of a pathogen, no significant changes in the expression, distribution, or labeling of RAB-10 were observed in NPR-17- and NPR-30-deficient cells (Fig.S8F-F’ and S8G), and the punctate accumulation of RAB-10 due to *P*. *aeruginosa* infection still occurred (Fig. S8F and S8Fʹ). These results suggest that opioid receptors in intestinal cells are unlikely to be the mechanism for sensing pathogen infection and causing changes in RAB-10 activity. Instead, the potential interaction between opioid receptors and RAB-10/Rab10 could be involved in regulating other biological processes, such as the neural and behavioral mechanisms that have been previously noted (Blazie et al., 2017; Cheong et al., 2015).

## Discussion

Our comprehension of PI(4,5)P2 metabolic activity and p38 MAPK signaling has been confined to the plasma membrane for years. Nevertheless, our research has broadened this scope by proposing the concept of endosome-dependent p38 MAPK signaling. This new perspective accentuates not just the continual hydrolysis of PI(4,5)P2, but also its precise intracellular localization, thus furthering our understanding of the spatial and temporal organization of this process. In this way, our work illuminates the role of RAB-10 in controlling PMK-1/p38 MAPK innate immunity, which is mediated by NHR-25-induced LET-413/Erbin activation and subsequent endosomal recruitment of UNC-16/JIP3 and EGL-8/PLC-β (Fig. S9; Table S3 and S4). It is important to note that the phospholipase-mediated hydrolysis of PI(4,5)P2 yields two distinct signaling molecules: DAGs and IP3 (inositol 1,4,5-trisphosphate) (O’Donnell et al., 2018). In *C*. *elegans*, ITR-1 has been identified as an IP3 receptor, controlling the rhythm of muscle contractions and associated behaviors through the periodic release of calcium (Dal Santo et al., 1999). Of note, mutations in *egl-8* and *unc-16* have been found to lead to impaired egg laying and defecation, as well as disruptions in body contraction (Byrd et al., 2001; Miller et al., 1999). Likewise, a deficiency of RAB-10 has been observed to result in defective egg laying (Daniele et al., 2020). Together, these findings reinforce the vital role of RAB-10, UNC-16, and EGL-8 in the hydrolysis of PI(4,5)P2 and its respective physiological implications.

The constitutively active Arf6 consistently triggers PI4P-5 kinase activation, causing an abnormal increase in endosomal PI(4,5)P2 levels, which hinders sorting and subsequent endosomal recycling (Brown et al., 2001; Naslavsky et al., 2003). In *C*. *elegans*, RAB-10 can recruit CNT-1/ARF-6-GAP onto endosomes to inhibit ARF-6 activity, ensuring appropriate levels of PI(4,5)P2 in endosomes (Shi et al., 2012). Accordingly, the intestinal cells of animals lacking RAB-10 have a significant accumulation of PI(4,5)P2 in their endosomes (Shi et al., 2012). However, this phenotype cannot be entirely attributed to the excessive activation of ARF-6 (Shi et al., 2012). Therefore, it is reasonable to assume that RAB-10 also regulates the hydrolysis of PI(4,5)P2 on endosomes. Our experiments revealed that UNC-16 and EGL-8 cooperate in the RAB-10-mediated hydrolysis of endosomal PI(4,5)P2. Furthermore, in the absence of CNT-1 and either UNC-16 or EGL-8, the accumulation of Tubby-PH^(R332H)^ on endosomes is comparable to the amount observed in RAB-10-deficient animals. These findings explain the discrepancy between the increased synthesis of endosomal PI(4,5)P2 as a result of the excessive activation of PPK-1/PI4P-5 kinase and the accumulation of PI(4,5)P2 caused by RAB-10 deficiency (Shi et al., 2012). Moreover, these findings have enabled us to propose a synergistic regulatory model of PI(4,5)P2 on endosomes, with RAB-10 controlling both the synthesis and hydrolysis processes. This model is advantageous as it integrates anabolism and catabolism, thus sustaining the equilibrium of PI(4,5)P2 on sorting endosomes and facilitating the advancement of recycling transport (Brown et al., 2001; Naslavsky et al., 2003). Of note, we observed that the reduction in PI(4,5)P2 production due to ARF-6 deficiency had no significant effect on the animal’s resistance to pathogen infection (Fig. S1A), suggesting the existence of alternative mechanisms for sustaining the intracellular level of PI(4,5)P2. Previous research has shown that RAB-10 limits the abundance of PI(4,5)P2 on sorting endosomes (Shi et al., 2012), likely ensuring the prevalence of PI3P in the endosomal membrane. This lipid composition facilitates the recruitment of proteins responsible for orchestrating endosomal sorting and maturation during the initial phase of the recycling pathway (Brown et al., 2001; Chen et al., 2018; Naslavsky et al., 2003; Shi et al., 2012). In contrast, the function of ARF-6 is postulated to be in the recycling endosome downstream of sorting endosomes (late phase of the recycling pathway) (Chen et al., 2018). By promoting the synthesis of recycling endosomal PI(4,5)P2, ARF-6 facilitates the recruitment of PI(4,5)P2-binding proteins like EHBP-1, RME-1, and AMPH-1 to promote membrane deformation and fission for transporting cargo to the plasma membrane (Chen et al., 2018; Shi et al., 2012). Therefore, it is reasonable to propose that the ARF-6-mediated production of endosomal PI(4,5)P2 serves as one of multiple mechanisms for preserving intracellular membrane PI(4,5)P2 levels. Indeed, research has shown that the levels of PI(4,5)P2 on endosomes can be cooperatively regulated by evolutionarily conserved lipid transfer proteins PDZD-8 and TEX-2, as well as PI(4,5)P2 phosphatase OCRL-1 and UNC-26/synaptojanin (Jeyasimman et al., 2021). These diverse regulatory mechanisms are likely to synergistically uphold the homeostasis of endosomal PI(4,5)P2, aligning with its functional relevance in numerous cellular processes.

PI(4,5)P2 is abundant in the plasma membrane (De Matteis and Godi, 2004), while its precursor, PI(4)P, is mainly sourced from the Golgi apparatus and the plasma membrane (Dickson et al., 2016). PI(4,5)P2 plays a role in endocytosis by enlisting proteins such as AP-2, epsin, and dynamin, and is promptly degraded post-endocytosis (McMahon and Boucrot, 2011). The rapid breakdown of PI(4,5)P2 in the endosomal membrane triggers changes in membrane properties, leading to the recruitment of proteins that possess an affinity for PI3P to facilitate the sorting and transport of endocytosed cargoes (Chen et al., 2018). Occurring at a high frequency and persistently, endocytosis is a significant dynamic membrane process on the plasma membrane. Hence, the sorting endosome is positioned as a critical site for PI(4,5)P2 hydrolysis within the cell, leading to the generation of signaling molecules that are anticipated to significantly impact the p38 MAPK pathway. In support of this proposition, the survival of *rab-10*(*ycx126*) animals was compromised following exposure to *P*. *aeruginosa*. This finding contributes a complementary subcellular insight to innate immune responses. It is also noteworthy that p38 MAPK plays a crucial role in regulating the GDP dissociation inhibitor (GDI) to modulate endocytic transport (Cavalli et al., 2001). Furthermore, it influences endocytosis by phosphorylating the Rab5 effector and impacts endolysosomal fission (Mace et al., 2005; Wible et al., 2024). Taken together, these studies suggest a significant interplay between endocytic trafficking and the p38 MAPK pathway, highlighting the existence of mutual regulatory mechanisms that have more profound implications than currently appreciated.

Of note, following infection, *rab-10*(*ok1494*) mutants exhibited residual *irg-4*::*gfp* expression (Fig. 1B), implying that the innate immune response facilitated by endosomal membrane may engage pathways beyond RAB-10-mediated PI(4,5)P2 hydrolysis. Indeed, the immune response of *C*. *elegans* is notably influenced by the interaction between vesicle-associated UNC-16 and JNK-1 (Byrd et al., 2001; Ewbank, 2006). Moreover, evidence shows that the assembly of the GPCR-TAB-p38 complex on endosomal membrane is involved in the regulation of immune signaling via the p38 MAPK pathway (Burton and Grimsey, 2019; Grimsey et al., 2018). Additionally, the regulatory role of TBC-2/Rab-GAP in bacterial pathogen stress resistance has been established, involving mechanisms that encompass both DAF-16-dependent and independent pathways (Traa et al., 2023). Altogether, these observations suggest that endosomal membranes function as a critical platform for multiple mechanisms related to innate immune modulation. Given the influence of PI(4,5)P2 metabolism on endosomal membrane properties and subsequent effects on the positioning of endosomal proteins, it is reasonable to suggest that PI(4,5)P2 metabolism serves as a fundamental mechanism in endosome-associated innate immune responses, thereby shaping the efficacy of downstream p38 MAPK, GPCR, and DAF-16 signaling pathways.

JNK-interacting proteins (JIPs) are capable of binding to multiple kinases in the c-Jun N-terminal kinase (JNK) pathway, thus making them identifiable as scaffolds (Whitmarsh, 2006). Consistently, studies have demonstrated that JIP3 serves as a scaffolding protein in neurons, interacting with both the kinesin-1 and dynein motor complexes (Sun et al., 2011). Our research suggests that UNC-16 is responsible for the endosomal residency of EGL-8. Furthermore, structural prediction and biochemical experiments indicate that the homodimer formed by UNC-16 through its LZII domain could act as a scaffold, facilitating the recruitment of EGL-8 onto endosomes. However, we noticed that the colocalization between EGL-8::GFP^SC KI^ and wrmScarlet::RAB-10^SC KI^ was not statistically significant (Fig. 5F and 5Fʹ), suggesting that the recruitment of UNC-16 and EGL-8 by RAB-10 likely occurs in a sequential manner as opposed to the formation of a complex comprising all three proteins through UNC-16 scaffolding. To attain a more profound comprehension of the spatial relationships of these molecules, a cryo-electron tomography (cryo-ET) analysis within functional cellular contexts is essential.

Our research has revealed that the increased RAB-10 activity caused by *P*. *aeruginosa* infection is likely due to the augmented expression of LET-413, and that NHR-25, a nuclear receptor, is required for the upsurge of LET-413 in this process. Interestingly, studies conducted on *C*. *elegans* have revealed that methionine in its environment can modulate the bacterial methyl cycle, thereby providing substrates for the production of S-adenosyl methionine (SAM) (Ding et al., 2015). SAM is a methyl donor for the biosynthesis of phosphatidylcholine in the hypodermis of the worm, and certain phosphatidylcholine molecules activate NHR-25, thus restraining the expression of the *grl-21* gene associated with hedgehog signaling (Lin and Wang, 2017). Moreover, a recent study showed that changes in phosphatidylcholine within the secretory membrane compartments can activate immunity-linked genes in worms (Fanelli et al., 2023). These findings suggest that bacterial metabolites could activate NHR-25 by increasing the production of SAM in the intestine, thus facilitating the synthesis of phosphatidylcholine. To assess the veracity of this hypothesis, further investigation is necessary. Particular attention should be devoted to determining whether *P*. *aeruginosa* infection leads to increased SAM production in intestinal cells and whether this heightened SAM production facilitates the synthesis of phosphatidylcholine.

## Supplementary data

Supplementary data is available at *Protein & Cell* online at https://doi.org/10.1093/procel/pwae041.

pwae041_suppl_Supplementary_Figures_S1-S9

pwae041_suppl_Supplementary_Table_S1

pwae041_suppl_Supplementary_Table_S2

pwae041_suppl_Supplementary_Table_S3

pwae041_suppl_Supplementary_Table_S4

## Data Availability

All the data that support the findings of this study are present in the paper and its Supplementary Information files. In addition, source data is available with the paper.
