# Supplementary material for: Endosomal catabolism of phosphatidylinositol 4,5-bisphosphate is fundamental in building resilience against pathogens
Source: Protein Cell. 2024 Aug 1;16(3):161–87. doi: 10.1093/procel/pwae041 (PMC11891140; doi:10.1093/procel/pwae041)
Supplement: pwae041_suppl_Supplementary_Figures_S1-S9 [file pwae041_suppl_supplementary_figures_s1-s9.pdf]

**Figure S1. The reduced pathogen resistance is not likely due to the impaired anabolism of PI(4,5)P2 and clathrin-mediated endocytosis.**

**(A)** Survival of wild-type, *arf-6(tm1447)*, and *amph-1(tm1060)* animals exposed to *P. aeruginosa*. The p-values for the comparisons are indicated (log-rank test). Sample sizes and mean lifespan are shown in Table S2.

**(B-B')** Confocal images of wild-type and *rab-10(ok1494)* animals expressing the transcriptional reporter *irg-4p::gfp* exposed to the indicated conditions. Box and whiskers plots (n = 25 animals): 10th-90th percentile; dots, outliers; red midline, median of *wild-type* animals; boundaries, quartiles. The p-values for the comparisons are indicated (one-way ANOVA with Tukey's multiple comparisons test). Scale bar, 100 μm.

**(C)** Survival of wild-type and *dpy-23(RNAi)* animals exposed to *P. aeruginosa*. The p-values for the comparisons are indicated (log-rank test). Sample sizes and mean lifespan are shown in Table S2.

**(D)** A schematic representation of the adult worm intestine in confocal imaging is presented, with the imaging plane close to the basal membrane of the intestinal cells designated as "Top", and the imaging plane where the apical membrane and lumen can be seen labeled as "Middle" (deep cytosol).

**(E-F')** Screening of nine reported RAB-10 effectors using PI(4,5)P2 reporter Tubby-PH$^{(R332H)}$::GFP and DAG reporter NES::GFP::C1ab-PKD1$^{SC\ KI}$. Box and whiskers plots (n = 24 animals): 10th-90th percentile; dots, outliers; red midline, median of wild-type; boundaries, quartiles. The p-values for the comparisons are indicated (one-way ANOVA with Dunnett's multiple comparisons test). Scale bars, 10 μm.

**Figure S2. Loss of UNC-16 leads to the aggregation of sorting endosomal structures.**

**(A)** Western blot showing UNC-16 expression in wild-type and *unc-16(e109)* animals.

**(B)** The membrane-to-cytosol ratio (P/S) of RAB-10 in wild-type and *unc-16(e109)* animals was measured. RAB-10 in the supernatants and pellets was analyzed by western blotting using an anti-GFP antibody. The loading control was blotted with the anti-β actin antibody. The average P/S ratio was calculated by normalizing the intensity of the supernatant or pellet with the corresponding β-actin, and the ratios are presented beneath the blots.

**(C-C')** Confocal images showing the localization of RAB-5 in wild-type and *unc-16(e109)* animals. Box and whiskers plots (n = 24 regions): 10th-90th percentile; dots, outliers; red midline, median of wild-type; boundaries, quartiles. The p-value for the comparison is indicated (two-tailed, unpaired t-test). Scale bars: 10 µm.

**(D)** The membrane-to-cytosol ratio (P/S) of RAB-5 in wild-type and *unc-16(e109)* animals was measured. RAB-5 in the supernatants and pellets was analyzed by western blotting using an anti-GFP antibody. The loading control was blotted with the anti-β actin antibody. The average P/S ratio was calculated by normalizing the intensity of the supernatant or pellet with the corresponding β-actin, and the ratios are presented beneath the blots.

**(E')** Confocal images showing the colocalization (upper panels: Z-axis focal plane; lower panels: Y-axis focal plane) between UNC-16 and ARF-6::GFP or GFP::RAB-7. The DAPI channel (blue color) indicates broad-spectrum intestinal autofluorescence. Mander's coefficients were calculated using the Z-stack confocal slices. The data is the mean with 95% confidence intervals (n=9 animals). Scale bars: 10 µm.

**Figure S3. An absence of UNC-16 induces an upsurge in endosomal PI(4,5)P2.**

**(A-B')** Confocal images showing the localization of Tubby-PH$^{(R332H)}$::GFP and NES::GFP::C1ab-PKD1$^{SC\ KI}$ in wild-type, UNC-16::MC, or MC:RAB-10(Q68L) animals. Box and whiskers plots (n = 24 regions): 10th-90th percentile; dots, outliers; red midline, median of wild-type; boundaries, quartiles. The p-values for the comparison are indicated (two-tailed, unpaired t-test). Scale bars: 10 µm.

**(C)** Survival of wild-type, UNC-16::MC, and MC:RAB-10(Q68L) animals exposed to *P. aeruginosa*. The p-values for the comparisons are indicated (log-rank test). Sample sizes and mean lifespan are shown in Table S2.

**(D-E')** Confocal images showing the localization of Tubby-PH$^{(R332H)}$::GFP and GFP::RME-1 in wild-type, *unc-16(e109)*, *arf-16(tm1447)*, and *arf-16(tm1447)*;*unc-16(e109)* animals. Box and whiskers plots (n = 24 regions): 10th-90th percentile; dots, outliers; red midline, median of wild-type; boundaries, quartiles. The p-values for the comparison are indicated (one-way ANOVA with Tukey's multiple comparisons test). Scale bars: 10 μm.

**(F)** The occupancy of wild-type, *rab-10(ok1494)*, and *unc-16(e109)* animals on a lawn of *P. aeruginosa* was measured. The data points represent the average of three replicates, and the error bars indicate 95% confidence intervals. The p-values for the comparisons are shown (one-way ANOVA with Sidak's multiple comparisons test).

**Figure S4. EGL-8/PLC-β is essential for the hydrolysis of endosomal PI(4,5)P2**

**(A-C')** Confocal images showing the localization of Tubby-PH$^{(R332H)}$::GFP, hTAC::GFP, and NES::GFP::C1ab-PKD1$^{SC\ KI}$ within cells after RNAi-mediated knockdown of 13 phospholipases, with *unc-16(RNAi)* as the phenotypical reference. Box and whiskers plots (n = 24 regions): 10th-90th percentile; dots, outliers; red midline, median of wild-type; boundaries, quartiles. The p-values for the comparison are indicated (one-way ANOVA with Dunnett's multiple comparisons test). Scale bars: 10 μm.

**Figure S5. EGL-8/PLC-β specifically interacts with UNC-16 and is involved in the hydrolysis of endosomal PI(4,5)P2.**

**(A)** Co-immunoprecipitation assays were performed using animals expressing epitope-tagged EGL-8 and RAB-10. IB, immunoblot; IP, immunoprecipitation; MC, mCherry.

**(B)** Western blot showing the interaction between RAB-10, loaded with either GTPγS

or GDP, and *in vitro* translated HA-tagged EGL-8.

**(C)** Western blot showing GST pull-down with *in vitro* translated HA-tagged PLC-1, PLC-2, PLC-3, and PLC-4.

**(D-D')** Confocal images showing the localization of Tubby-PH$^{(R332H)}$::GFP in wild-type, *unc-16(e109)*, *unc-16(e109)*;EGL-8::MC(OE), *unc-16(e109)*;PLC-1::MC(OE), *unc-16(e109)*;PLC-2::MC(OE), *unc-16(e109)*;PLC-3::MC(OE), and *unc-16(e109)*;PLC-4::MC(OE) animals. Box and whiskers plots (n = 24 regions): 10th-90th percentile; dots, outliers; red midline, median of wild-type; boundaries, quartiles. The p-values for the comparison are indicated (one-way ANOVA with Dunnett's multiple comparisons test). MC, mCherry. Scale bars: 10 μm.

**Figure S6. EGL-8 is essential for the RAB-10-mediated innate immune response.**

**(A)** Survival of wild-type, *rab-10(ok1494)*, and *rab-10(ok1494)*;EGL-8::mCherry(OE) animals was examined when exposed to *P. aeruginosa.* The p-values for the comparisons are indicated (log-rank test). Sample sizes and mean lifespan are shown in Table S2.

**(B-B')** Confocal images of wild-type, *rab-10(ok1494)*, and *rab-10(ok1494)*;EGL-8::mCherry(OE) animals expressing the transcriptional reporter *irg-4p::gfp* after exposure to *P. aeruginosa*. Box and whiskers plots (n = 25 animals): 10th-90th percentile; dots, outliers; red midline, median of *wild-type* animals; boundaries, quartiles. The p-values for the comparisons are indicated (one-way ANOVA with Tukey's multiple comparisons test). Scale bar, 100 μm.

**Figure S7. Overexpression of UNC-16(L360P) failed to restore the subcellular localization of Tubby-PH$^{(R332H)}$, hTAC::GFP, and EGL-8::GFP$^{SC\ KI}$ in UNC-16-deficient animals, and the mRNA level of TBC-11 was not affected by *P. aeruginosa* infection.**

**(A-C')** Confocal images showing the localization of Tubby-PH$^{(R332H)}$::GFP, hTAC::GFP, and EGL-8::GFP$^{SC\ KI}$ in wild-type, *unc-16(e109)*, *unc-16(e109)*;UNC-16::mCherry(OE), and *unc-16(e109)*;UNC-16(L360P)::mCherry(OE) animals. Box and whiskers plots (n = 24 regions): 10th-90th percentile; dots, outliers; red midline, median of wild-type; boundaries, quartiles. The p-values for the comparison are indicated (one-way ANOVA with Dunnett's multiple comparisons test). Scale bars: 10 μm.

**(D)** qPCR analysis evaluating the expression of *tbc-11* before and after exposure to *P. aeruginosa*. The data were the mean ± SEM of three replicates. The p-value for the comparison is indicated (two-tailed, paired t-test).

**Figure S8. The upregulation of LET-413 in response to *P. aeruginosa* infection is contingent upon the presence of NHR-25.**

**(A-A')** Confocal images showing the localization of endogenous UNC-16 in the absence or presence of *P. aeruginosa*. Box and whiskers plots (n = 24 regions): 10th-90th percentile; dots, outliers; red midline, median of wild-type; boundaries, quartiles. The p-value for the comparison is indicated (two-tailed, unpaired t-test). Scale bars, 10 μm.

**(B-B')** Western blot showing the endogenous levels of UNC-16. The relative intensity of UNC-16 was quantified relative to actin. The data were the mean ± SEM of three replicates. The p-value for the comparison is indicated (two-tailed, paired t-test).

**(C-C')** Confocal images showing the localization of EGL-8::GFP$^{SC\ KI}$ in the absence or presence of *P. aeruginosa*. Box and whiskers plots (n = 24 regions): 10th-90th percentile; dots, outliers; red midline, median of wild-type; boundaries, quartiles. The p-value for the comparison is indicated (two-tailed, unpaired t-test). Scale bars, 10 μm.

**(D-D')** Western blot showing the endogenous levels of EGL-8. The relative intensity of EGL-8 was quantified relative to actin. The data were the mean ± SEM of three replicates. The p-value for the comparison is indicated (two-tailed, paired t-test).

**(E)** qPCR analysis evaluating the expression of *unc-16* and *egl-8* before and after exposure to *P. aeruginosa*. The data were the mean ± SEM of three replicates. The p-values for the comparison are indicated (one-way ANOVA with Dunnett'smultiple comparisons test).

**(F-F')** Confocal images showing the localization of endogenous RAB-10 in the absence or presence of *P. aeruginosa*. Box and whiskers plots (n = 24 regions): 10th-90th percentile; dots, outliers; red midline, median of wild-type; boundaries, quartiles. The p-values for the comparison are indicated (one-way ANOVA with Dunnett's multiple comparisons test). Scale bars, 10 μm.

**(G)** Western blot showing the endogenous levels of RAB-10. The relative intensity of RAB-10 was quantified relative to actin. The ratios have been provided beneath the blots.

**Figure S9.** A model illustrating that in the intestine of *C. elegans*, RAB-10 mediates the hydrolysis of endosomal PI(4,5)P2, which is essential for developing immunity to pathogens. The amplified RAB-10 activity, due to *P. aeruginosa* infection, could be attributed to an increase in LET-413 expression, which is regulated by the nuclear receptor family transcription factor NHR-25.

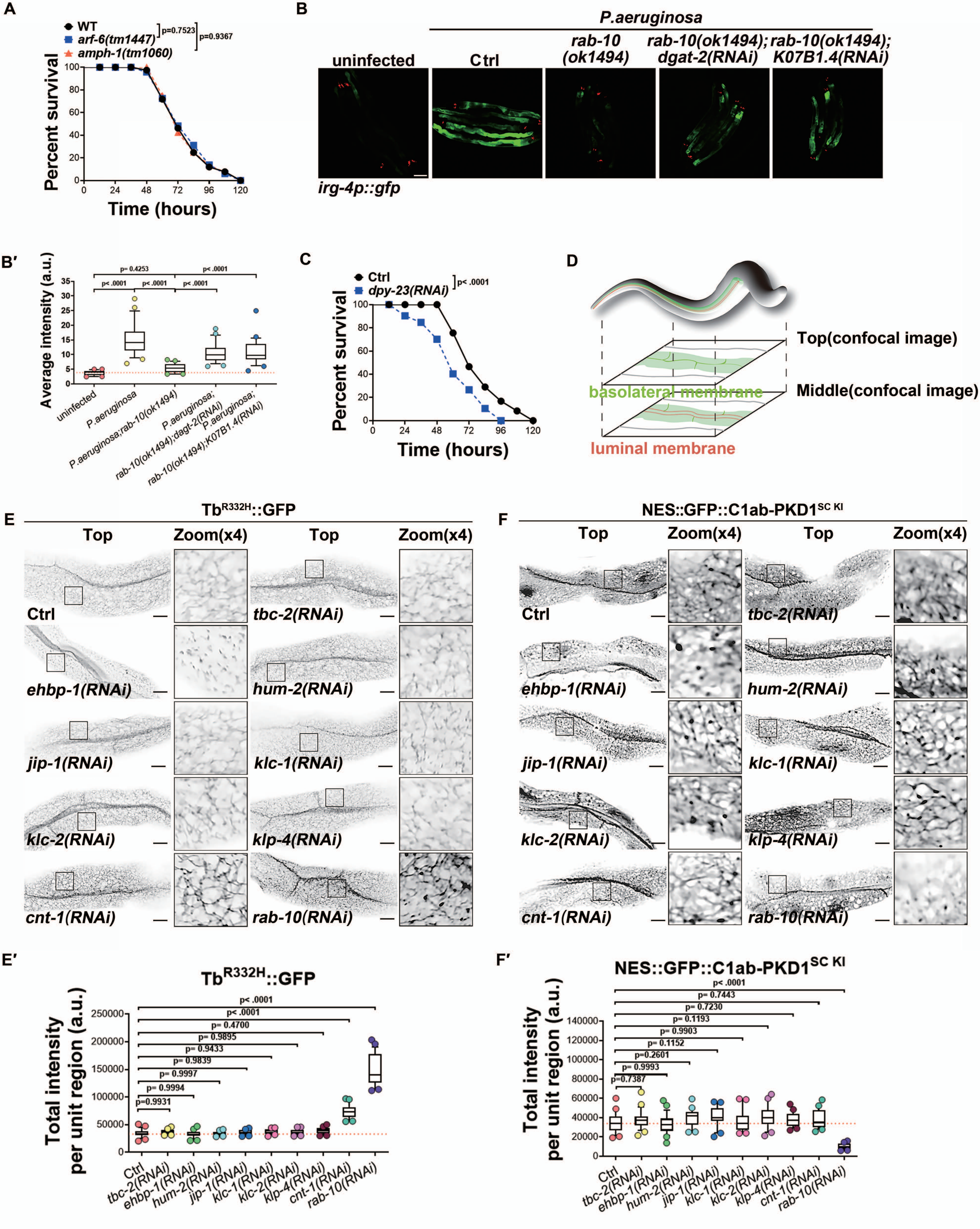

A
WT
arf-6(tm1447)
amph-1(tm1060)
p=0.7523
p=0.9367
Percent survival
Time (hours)
B
P.aeruginosa
uninfected
Ctrl
rab-10 (ok1494)
rab-10(ok1494); dgat-2(RNAi)
rab-10(ok1494); K07B1.4(RNAi)
irg-4p::gfp
B′
Average intensity (a.u.)
p= 0.4253
p< .0001
uninfected
P.aeruginosa
P.aeruginosa;rab-10(ok1494)
P.aeruginosa; rab-10(ok1494);dagt-2(RNAi)
P.aeruginosa; rab-10(ok1494);K07B1.4(RNAi)
C
Ctrl
dpy-23(RNAi)
p< .0001
Percent survival
Time (hours)
D
Top(confocal image)
Middle(confocal image)
basolateral membrane
luminal membrane
E
Tb^R332H::GFP
Top
Zoom(x4)
Ctrl
tbc-2(RNAi)
ehbp-1(RNAi)
hum-2(RNAi)
jip-1(RNAi)
klc-1(RNAi)
klc-2(RNAi)
klp-4(RNAi)
cnt-1(RNAi)
rab-10(RNAi)
F
NES::GFP::C1ab-PKD1^SC KI
Top
Zoom(x4)
Ctrl
tbc-2(RNAi)
ehbp-1(RNAi)
hum-2(RNAi)
jip-1(RNAi)
klc-1(RNAi)
klc-2(RNAi)
klp-4(RNAi)
cnt-1(RNAi)
rab-10(RNAi)
E′
Tb^R332H::GFP
Total intensity per unit region (a.u.)
p< .0001
p< .0001
p= 0.4700
p= 0.9895
p= 0.9433
p= 0.9839
p= 0.9997
p= 0.9994
p=0.9931
Ctrl
tbc-2(RNAi)
ehbp-1(RNAi)
hum-2(RNAi)
jip-1(RNAi)
klc-1(RNAi)
klc-2(RNAi)
klp-4(RNAi)
cnt-1(RNAi)
rab-10(RNAi)
F′
NES::GFP::C1ab-PKD1^SC KI
Total intensity per unit region (a.u.)
p< .0001
p= 0.7443
p= 0.7230
p= 0.1193
p= 0.9903
p= 0.1152
p=0.2601
p= 0.9993
p=0.7387
Ctrl
tbc-2(RNAi)
ehbp-1(RNAi)
hum-2(RNAi)
jip-1(RNAi)
klc-1(RNAi)
klc-2(RNAi)
klp-4(RNAi)
cnt-1(RNAi)
rab-10(RNAi)

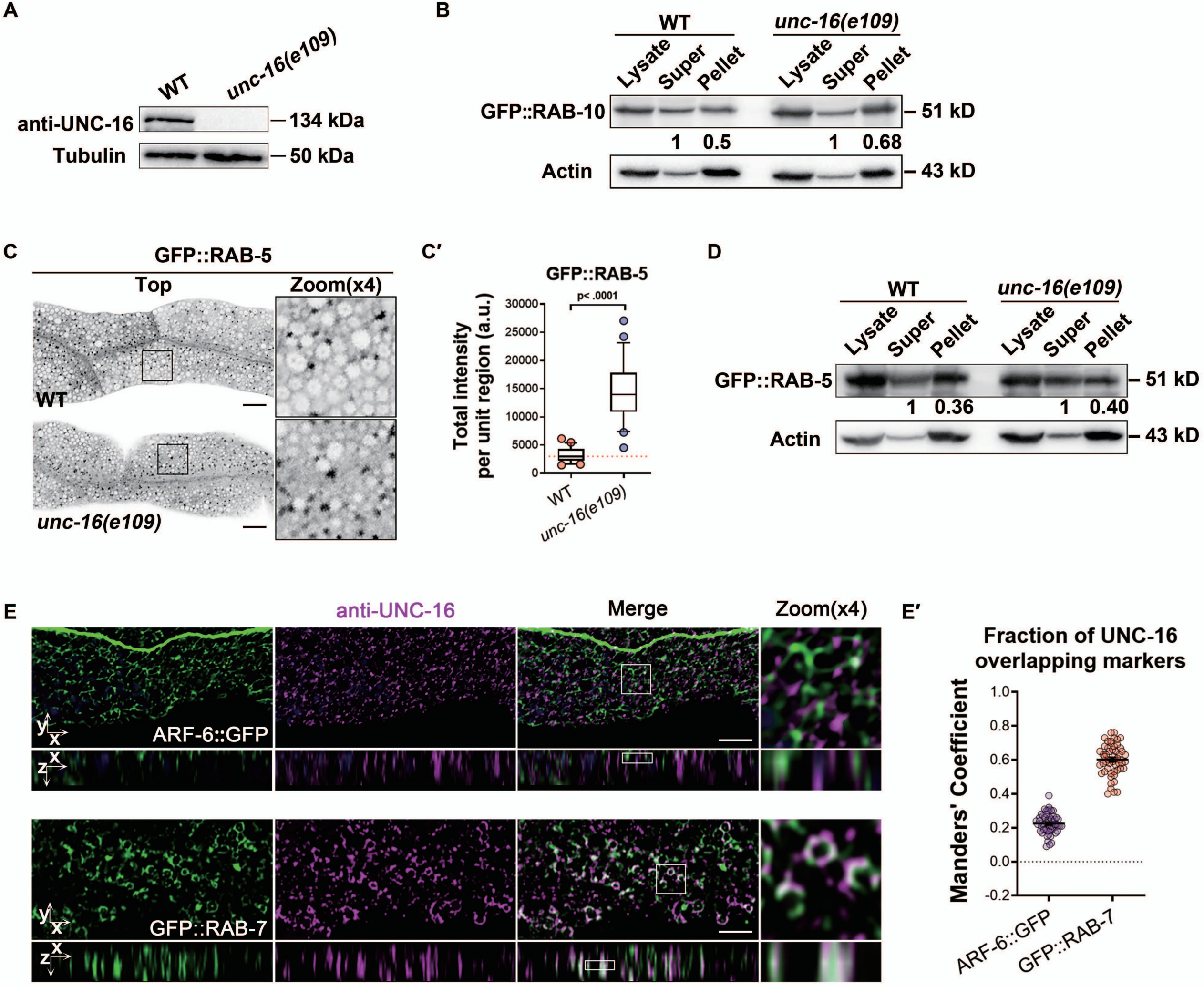

A
WT
unc-16(e109)
anti-UNC-16
134 kDa
Tubulin
50 kDa
B
WT
unc-16(e109)
Lysate
Super
Pellet
GFP::RAB-10
51 kD
1
0.5
0.68
Actin
43 kD
C
GFP::RAB-5
Top
Zoom(x4)
C′
Total intensity per unit region (a.u.)
p< .0001
D
GFP::RAB-5
0.36
0.40
E
anti-UNC-16
Merge
ARF-6::GFP
GFP::RAB-7
E′
Fraction of UNC-16 overlapping markers
Manders' Coefficient

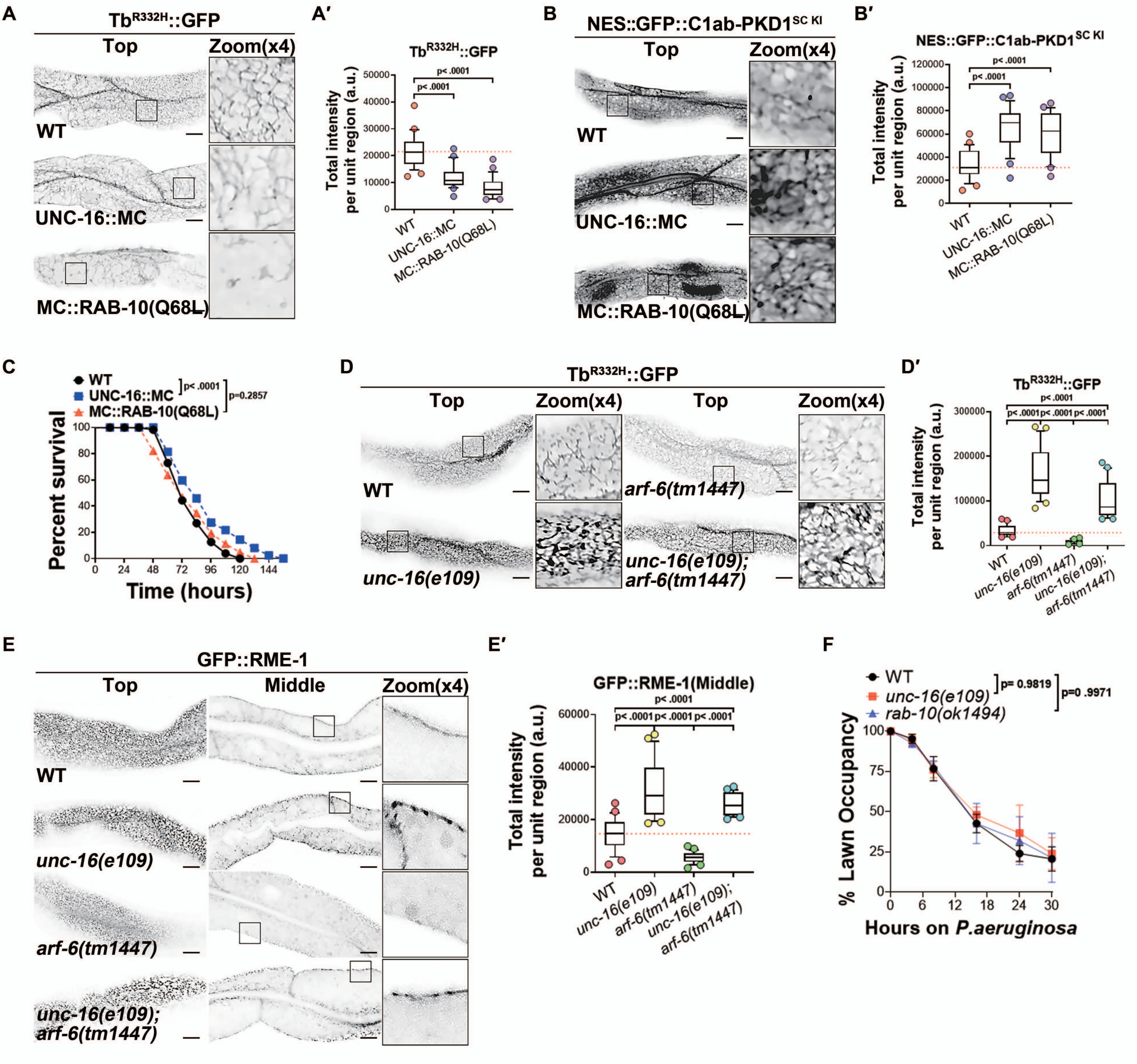

A
Tb^R332H::GFP
Top
Zoom(x4)
WT
UNC-16::MC
MC::RAB-10(Q68L)
A′
Tb^R332H::GFP
Total intensity per unit region (a.u.)
p< .0001
p< .0001
B
NES::GFP::C1ab-PKD1^SC KI
B′
NES::GFP::C1ab-PKD1^SC KI
C
WT
UNC-16::MC
MC::RAB-10(Q68L)
p< .0001
p=0.2857
Percent survival
Time (hours)
D
Tb^R332H::GFP
unc-16(e109)
arf-6(tm1447)
unc-16(e109); arf-6(tm1447)
D′
Tb^R332H::GFP
E
GFP::RME-1
Middle
E′
GFP::RME-1(Middle)
F
WT
unc-16(e109)
rab-10(ok1494)
p= 0.9819
p=0 .9971
% Lawn Occupancy
Hours on P.aeruginosa

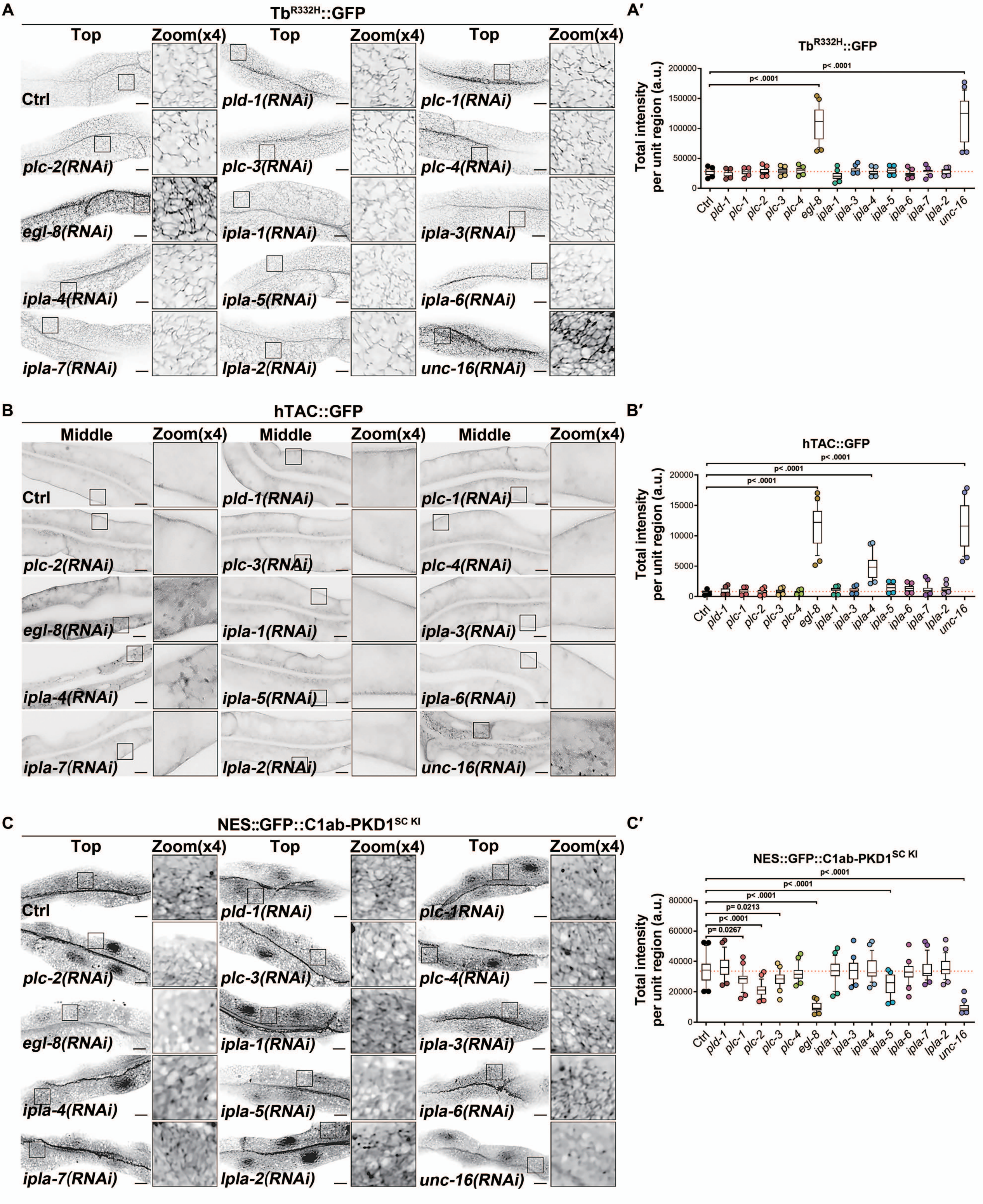
A
Tb^R332H::GFP
Top
Zoom(x4)
Ctrl
pld-1(RNAi)
plc-1(RNAi)
plc-2(RNAi)
plc-3(RNAi)
plc-4(RNAi)
egl-8(RNAi)
ipla-1(RNAi)
ipla-3(RNAi)
ipla-4(RNAi)
ipla-5(RNAi)
ipla-6(RNAi)
ipla-7(RNAi)
lpla-2(RNAi)
unc-16(RNAi)
A′
Tb^R332H::GFP
Total intensity per unit region (a.u.)
p< .0001
p< .0001
200000
150000
100000
50000
0
Ctrl pld-1 plc-1 plc-2 plc-3 plc-4 egl-8 ipla-1 ipla-3 ipla-4 ipla-5 ipla-6 ipla-7 lpla-2 unc-16
B
hTAC::GFP
Middle
Zoom(x4)
Ctrl
pld-1(RNAi)
plc-1(RNAi)
plc-2(RNAi)
plc-3(RNAi)
plc-4(RNAi)
egl-8(RNAi)
ipla-1(RNAi)
ipla-3(RNAi)
ipla-4(RNAi)
ipla-5(RNAi)
ipla-6(RNAi)
ipla-7(RNAi)
lpla-2(RNAi)
unc-16(RNAi)
B′
hTAC::GFP
Total intensity per unit region (a.u.)
p< .0001
p< .0001
p< .0001
20000
15000
10000
5000
0
Ctrl pld-1 plc-1 plc-2 plc-3 plc-4 egl-8 ipla-1 ipla-3 ipla-4 ipla-5 ipla-6 ipla-7 lpla-2 unc-16
C
NES::GFP::C1ab-PKD1^SC KI
Top
Zoom(x4)
Ctrl
pld-1(RNAi)
plc-1RNAi)
plc-2(RNAi)
plc-3(RNAi)
plc-4(RNAi)
egl-8(RNAi)
ipla-1(RNAi)
ipla-3(RNAi)
ipla-4(RNAi)
ipla-5(RNAi)
ipla-6(RNAi)
ipla-7(RNAi)
lpla-2(RNAi)
unc-16(RNAi)
C′
NES::GFP::C1ab-PKD1^SC KI
Total intensity per unit region (a.u.)
p< .0001
p< .0001
p< .0001
p= 0.0213
p< .0001
p= 0.0267
80000
60000
40000
20000
0
Ctrl pld-1 plc-1 plc-2 plc-3 plc-4 egl-8 ipla-1 ipla-3 ipla-4 ipla-5 ipla-6 ipla-7 lpla-2 unc-16

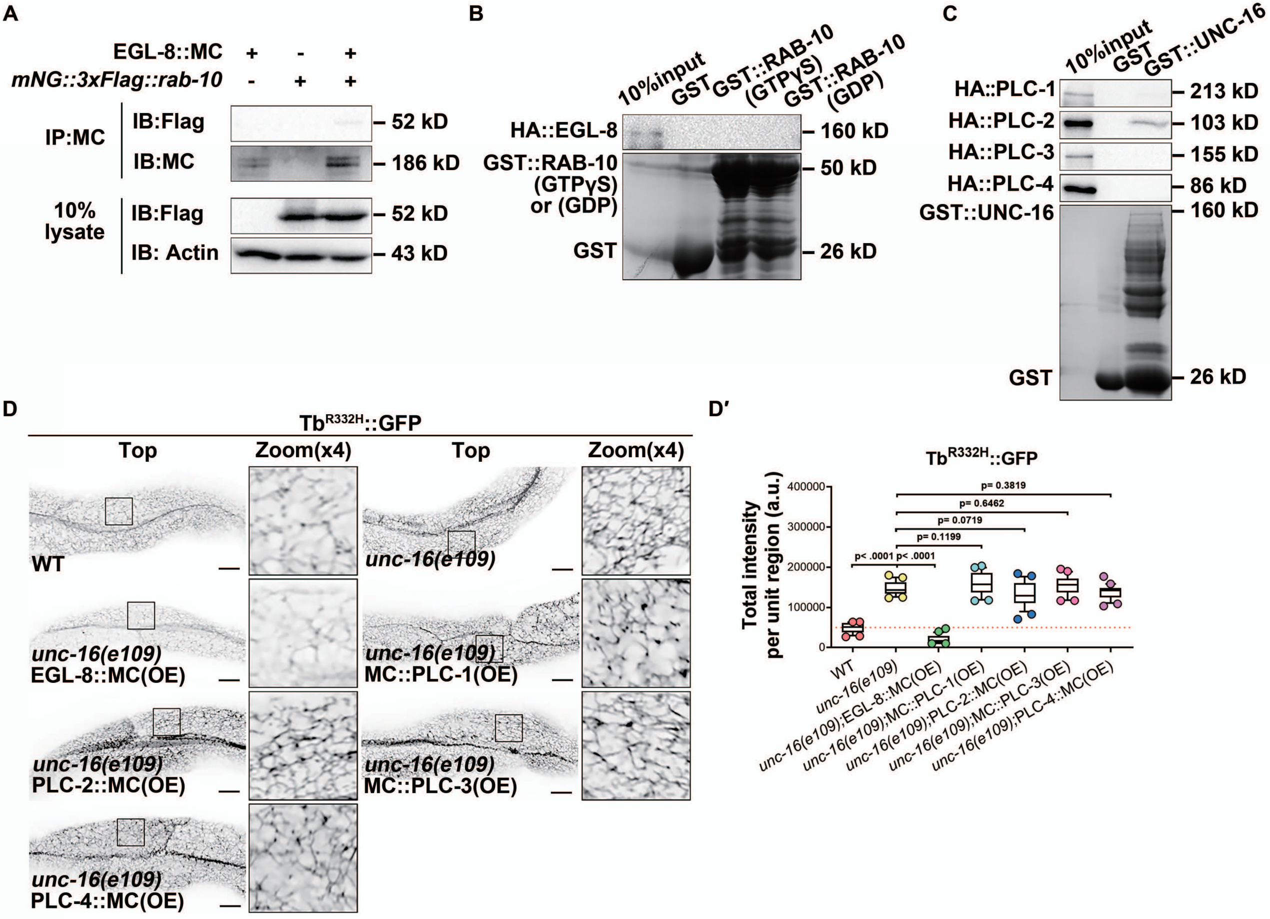
A
EGL-8::MC + - +
mNG::3xFlag::rab-10 - + +
IP:MC
IB:Flag
IB:MC
10% lysate
IB:Flag
IB: Actin
52 kD
186 kD
52 kD
43 kD
B
10%input
GST
GST::RAB-10 (GTPγS)
GST::RAB-10 (GDP)
HA::EGL-8
GST::RAB-10 (GTPγS) or (GDP)
GST
160 kD
50 kD
26 kD
C
10%input
GST
GST::UNC-16
HA::PLC-1
HA::PLC-2
HA::PLC-3
HA::PLC-4
GST::UNC-16
GST
213 kD
103 kD
155 kD
86 kD
160 kD
26 kD
D
Tb^R332H::GFP
Top
Zoom(x4)
WT
unc-16(e109)
unc-16(e109) EGL-8::MC(OE)
unc-16(e109) MC::PLC-1(OE)
unc-16(e109) PLC-2::MC(OE)
unc-16(e109) MC::PLC-3(OE)
unc-16(e109) PLC-4::MC(OE)
D′
Tb^R332H::GFP
Total intensity per unit region (a.u.)
400000
300000
200000
100000
0
p= 0.3819
p= 0.6462
p= 0.0719
p= 0.1199
p< .0001
p< .0001
WT
unc-16(e109)
unc-16(e109);EGL-8::MC(OE)
unc-16(e109);MC::PLC-1(OE)
unc-16(e109);PLC-2::MC(OE)
unc-16(e109);MC::PLC-3(OE)
unc-16(e109);PLC-4::MC(OE)
